# Inhibition of soluble epoxide hydrolase confers neuroprotection and restores microglial homeostasis in a tauopathy mouse model

**DOI:** 10.1186/s13024-025-00844-x

**Published:** 2025-04-23

**Authors:** Shuo Wang, Chuangye Qi, Chetan Rajpurohit, Baijayanti Ghosh, Wen Xiong, Baiping Wang, Yanyan Qi, Sung Hee Hwang, Bruce D. Hammock, Hongjie Li, Li Gan, Hui Zheng

**Affiliations:** 1 Huffington Center on Aging, Baylor College of Medicine, One Baylor Plaza, Houston, TX 77030 USA; 2 Department of Molecular and Human Genetics, Baylor College of Medicine, Houston, TX USA; 3 Department of Entomology and Nematology and UC Davis Comprehensive Cancer Center, University of California, Davis, CA USA; 4 Helen and Robert Appel Alzheimer’s Disease Research Institute, Weill Cornell Medicine, New York, NY USA

**Keywords:** Alzheimer’s disease, Epoxyeicosatrienoic acids, Microglia, Neurodegeneration, Soluble epoxide hydrolase, Tau

## Abstract

**Background:**

The epoxyeicosatrienoic acids (EETs) are derivatives of the arachidonic acid metabolism with anti-inflammatory activities. However, their efficacy is limited due to the rapid hydrolysis by soluble epoxide hydrolase (sEH). Inhibition of sEH has been shown to stabilize the EETs and reduce neuroinflammation in Aβ mouse models of Alzheimer’s disease (AD). However, the role of the sEH-EET signaling pathway in other CNS cell types and neurodegenerative conditions are less understood.

**Methods:**

Here we investigated the mechanisms and functional role of the sEH-EET axis in tauopathy by treating PS19 mice with a small molecule sEH inhibitor TPPU and by crossing the PS19 mice with *Ephx2* (gene encoding sEH) knockout mice. This was followed by single-nucleus RNA-sequencing (snRNA-seq), biochemical and immunohistochemical analysis, and behavioral assessments. Additionally, we examined the effects of the sEH-EET pathway in primary microglia cultures and human induced pluripotent stem cell (iPSC)-derived neurons exhibiting seeding-induced Tau inclusions.

**Results:**

sEH inhibition improved cognitive function, rescued neuronal cell loss, and reduced Tau pathology and microglial reactivity. snRNA-seq revealed that TPPU treatment upregulated genes involved in actin cytoskeleton and excitatory synaptic pathways. Treatment of human iPSC-derived neurons with TPPU enhanced synaptic density without affecting Tau accumulation, suggesting a cell-autonomous neuroprotective effect of sEH blockade. Furthermore, sEH inhibition reversed disease-associated and interferon-responsive microglial states in PS19 mice, while EET supplementation promoted Tau phagocytosis and clearance in primary microglia cultures.

**Conclusion:**

These findings demonstrate that sEH blockade or EET augmentation confers therapeutic benefit in neurodegenerative tauopathies by simultaneously targeting neuronal and microglial pathways.

**Supplementary Information:**

The online version contains supplementary material available at 10.1186/s13024-025-00844-x.

## Background

Alzheimer’s Disease (AD) is a progressive neurodegenerative disorder manifested by the deposition of beta amyloid (Aβ) plaques, the accumulation of neurofibrillary tangles (NFTs) composed of aggregated Tau protein, and extensive neurodegeneration. These pathological events are accompanied by the hyperactivation of microglia and astrocytes, along with chronic neuroinflammation [1]. Although anti-Aβ antibodies have shown clinical efficacy in prodromal AD [2, 3], NFTs correlates more closely with cognitive decline than Aβ plaques [4]. Therefore, anti-Aβ therapies may not be effective in the late stages when Tau pathology and neuroinflammation ensue. Additionally, NFTs are a pathological feature of a group of diseases collectively termed tauopathies and can cause neurodegeneration in and of itself [5, 6]. Thus, there is strong rationale for developing therapies targeting Tau and neuroinflammatory pathways for both AD and broader tauopathy conditions [1, 7, 8].

Arachidonic acid is a polyunsaturated fatty acid released from the membrane phospholipids, which can be further metabolized to epoxyeicosatrienoic acids (EETs) by cytochrome P450 enzymes (CYPs) [9]. EETs have demonstrated anti-inflammatory and inflammation-resolving properties [10]. However, their efficacy is limited due to the rapid hydrolysis by the soluble epoxide hydrolase (sEH). sEH, encoded by the *EPHX2* gene, is widely expressed in both peripheral tissues and central nervous system (CNS) [11, 12]. Elevated sEH levels have been implicated in neurovascular and neurological conditions such as vascular cognitive impairment [13], depression [14], schizophrenia [15], and Parkinson’s disease [16].

Multiple lines of evidence support a direct role of sEH in AD. Specifically, *EPHX2* is located within the *PTK2B-CLU* AD risk locus in chromosome 8, where a disease-associated polymorphism has been reported to influence its expression [17, 18]. Proteome-wide association studies also revealed a potential link of EPHX2/sEH with AD [19, 20]. Furthermore, targeted lipidomic analyses of brain lysates, plasma and cerebrospinal fluid samples have identified dysregulation of CYP and sEH metabolic pathways in AD [21, 22]. Consistent with human data, inhibition of sEH—either genetically or pharmacologically—has been shown to elevate EET levels and reduce Aβ pathology in mouse models [23–25]. While these effects have largely been attributed to the immunomodulatory functions of EETs on glial cells, particularly microglia, the role of the sEH-EET axis in other cell types such as neurons are poorly understood. Additionally, a possible role for sEH inhibition in Tau pathogenesis has not been reported.

Here we treated the PS19 Tau P301S transgenic mice, which develop age-dependent pathological Tau accumulation and neuronal cell loss, with a selective sEH inhibitor, 1-trifluoromethoxyphenyl-3-(1-propionylpiperidin-4-yl) urea (TPPU) [11, 14]. We also generated *Ephx2*-null PS19 mice by crossing the *Ephx2* knockout mice with PS19 mice [26]. We show that both genetic ablation and pharmacological inhibition of sEH reduced microglial reactivity, attenuated Tau pathology and rescued neurodegeneration and cognitive impairment. Single nucleus RNA-sequencing (snRNA-seq) revealed microglia and dentate granule cells as the major cell types altered in PS19 mice, changes that were reversed by TPPU treatment. Using primary microglia cultures and human induced pluripotent stem cell (iPSC)-derived neurons [27], we demonstrate a direct effect of neuronal sEH inhibition in promoting synaptic health, while EET augmentation enhanced microglial phagocytosis and Tau clearance.

## Methods

### Study design

The goal of this study was to determine the role of the sEH-EET epoxy lipid signaling pathway in diseases of tauopathy including AD, using a small molecule sEH inhibitor TPPU or by genetic ablation of *Ephx2* in PS19 mice. Additionally, we treated the iPSC neurons or primary microglia cultures with TPPU or EET, respectively, to evaluate the mechanisms. The animals, plates, and slides were randomized prior to the experimentation and were blinded to the experimenters. The minimum number of mice for all experiments was at least six per group based on our previous studies [23, 28]. The exact number of mice are listed in the figure legends and marked on the bar graph figures. All in vitro experiments were performed at least twice, each with at least three technical repeats. Additional experimental details are described in this section and in figure legends.

**Fig. 1:**
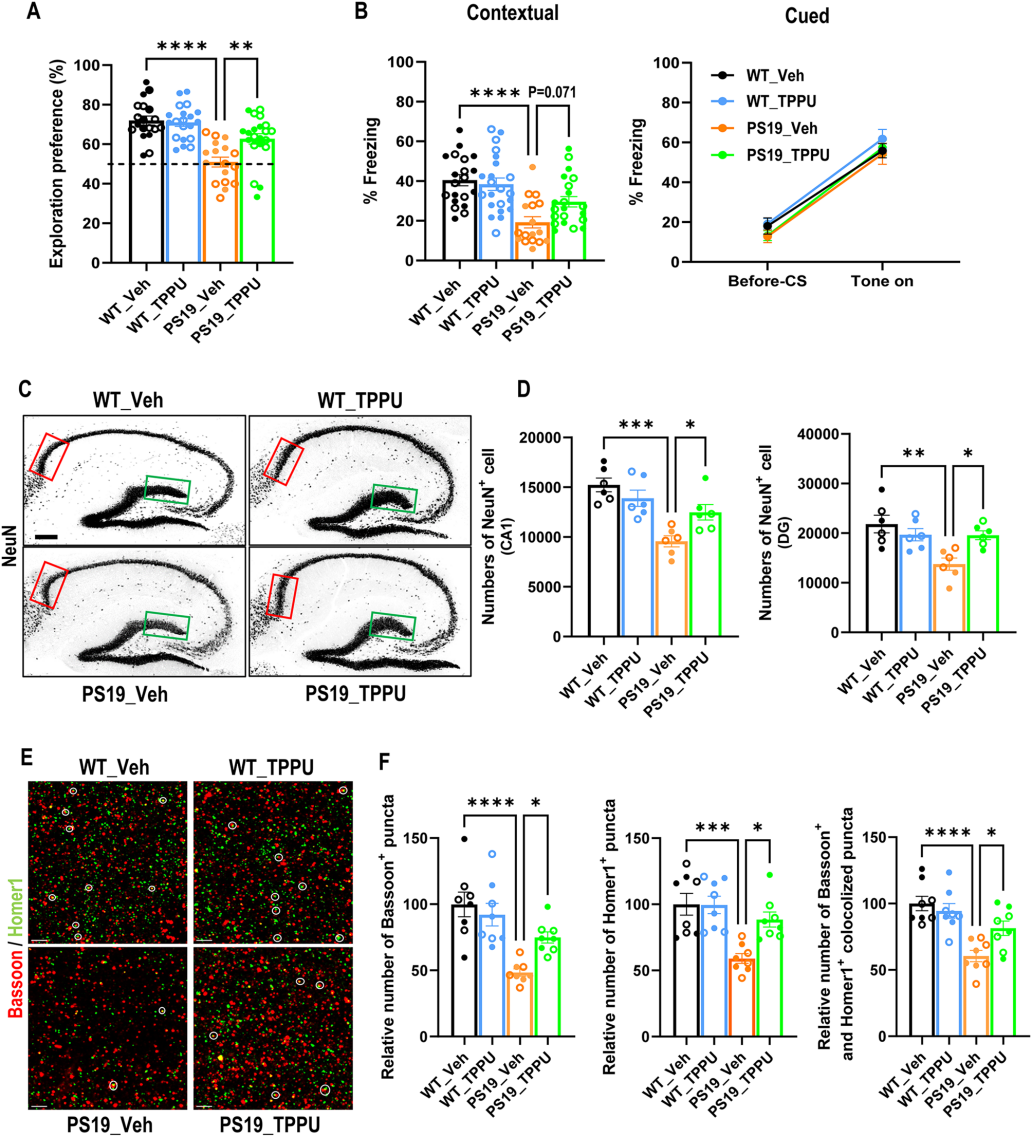
sEH inhibition rescues neuronal and synapse loss and improves cognitive function in PS19 mice. **(A)** Quantification of percentage time exploring the novel object in the novel object recognition test performed in 9-9.5-month-old mice. The dashed line represents a 50% chance of random object exploration. Wild-type mice with vehicle treatment (WT_Veh): *n* = 10♂ and 9♀; Wild-type mice with TPPU treatment (WT_TPPU): *n* = 12♂ and 9♀; PS19 mice with vehicle treatment (PS19_Veh): *n* = 8♂and 9♀; and PS19 mice with TPPU treatment (PS19_TPPU): *n* = 10 ♂and 11♀. **(B)** Contextual (left) and cued (right) fear conditioning test performed in 9-9.5-month-old mice. WT_Veh: *n* = 11♂ and 9♀; WT_TPPU: *n* = 12 ♂and 9♀; PS19_Veh: *n* = 8♂ and 9♀; PS19_TPPU: *n* = 10♂ and 11♀. **(C)** Representative NeuN immunofluorescence staining images in hippocampus of WT_Veh, WT_TPPU, PS19_Veh, and PS19_TPPU mice at 9.5–10 months. Rectangles mark CA1 (red) and dentate gyrus (DG; green) areas selected for quantification. Scale bar: 200 μm. **(D)** Estimate of neuronal numbers in CA1 (left) and DG (right) using unbiased stereology. *n* = 3♂ and 3♀ per group. **(E)** Representative images of presynaptic marker Bassoon (red) and postsynaptic marker Homer1 (green) immunofluorescence staining in CA1 area of hippocampus from 9.5-10-month-old mice. Colocalized puncta are marked by white circles. Scale bar: 3 µm. **(F)** Quantification of relative number of Bassoon^+^ puncta, Homer1^+^ puncta and colocalized Bassoon^+^ and Homer1^+^ puncta (percentage of WT_Veh). *n* = 4♂ and 4♀ per group. Filled circle: ♂; open circle: ♀. Data are presented as mean ± SEM. One-way ANOVA with Tukey’s multiple comparison test. *p < 0.05; **p < 0.01; ***p < 0.001; ****p < 0.0001. See also Figures S2 and S3

### Human subjects

Postmortem brain tissues were provided by the University of Pennsylvania Center for Neurodegenerative Disease Research. Informed consent was obtained from all subjects. The demographic data can be found in Supplemental Table 1. The influence of gender was not analyzed due to small sample size.

**Fig. 2:**
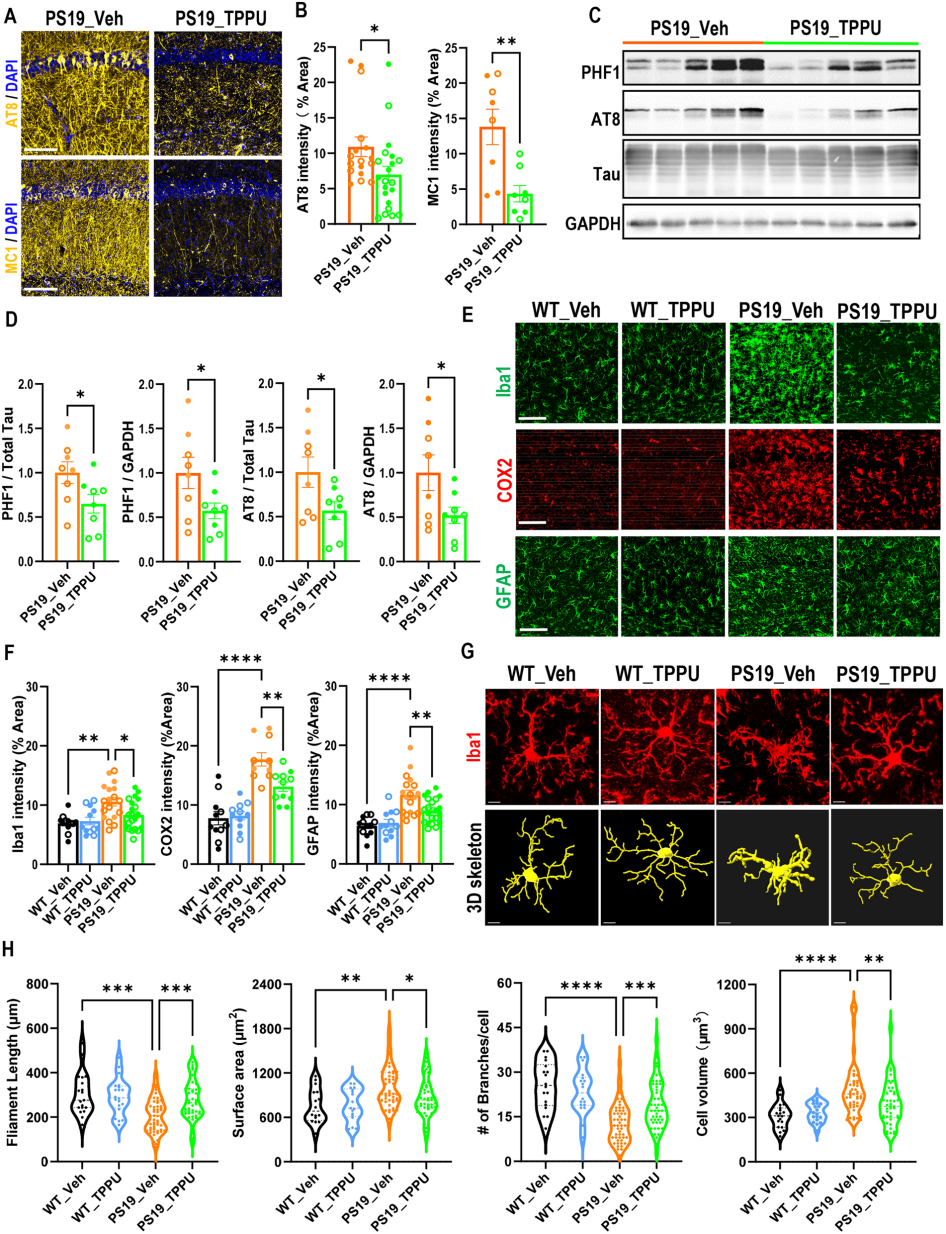
sEH inhibition attenuated Tau pathology and reactive gliosis in PS19 mice. **(A)** Representative immunofluorescence staining of hippocampal CA1 region of PS19_Veh and PS19_TPPU mice with AT8 and MC1 antibodies. Scale bar: 100 μm. **(B)** Quantification of AT8 and MC1 intensities. AT8: PS19_Veh: *n* = 8♂ and 9♀; PS19_TPPU: *n* = 10♂ and 11♀. MC1: PS19_Veh: *n* = 5♂ and 3♀; PS19_TPPU: *n* = 4♂ and 4♀. **(C)** Representative Western blots of PHF1- and AT8-positive phospho-Tau and total Tau in hippocampus of 9.5-10-month-old PS19_Veh and PS19_TPPU mice. GAPDH was used as a loading control. **(D)** Quantification of (C). *n* = 3♂and 5♀per group. **(E)** Representative Iba1, COX2 or GFAP immunofluorescence staining images in CA1 area of hippocampus of 9.5-10-month-old mice. Scale bar: 100 µm. **(F)** Quantification of Iba1, COX2 and GFAP staining intensity. Iba1/GFAP: WT_Veh: *n* = 7♂and 5♀; WT_TPPU: *n* = 7♂and 5♀; PS19_Veh: *n* = 8♂ and 10♀; PS19_TPPU: *n* = 10♂ and 11♀. COX2: WT_Veh: *n* = 6♂and 5♀; WT_TPPU: *n* = 6♂and 5♀; PS19_Veh: *n* = 4♂ and 6♀; PS19_TPPU: *n* = 5♂ and 6♀. **(G)** Representative Iba1 immunofluorescence staining images and 3D skeletonization of microglia in the CA1 area of hippocampus of 9.5-10-month-old mice. Scale bar: 7 µm. **(H)** Quantification of microglia filament length, surface area, number of branches and volume per cell using the IMARIS software. *n* = 3♂and 3♀ per group. Filled circle: ♂; open circle: ♀. Data are presented as mean ± SEM. B and D: Student *t*’s test; F and H: One-way ANOVA with Tukey’s multiple comparison test. *p < 0.05; **p < 0.01; ***p < 0.001; ****p < 0 0.0001. See also Figure S4

**Fig. 3:**
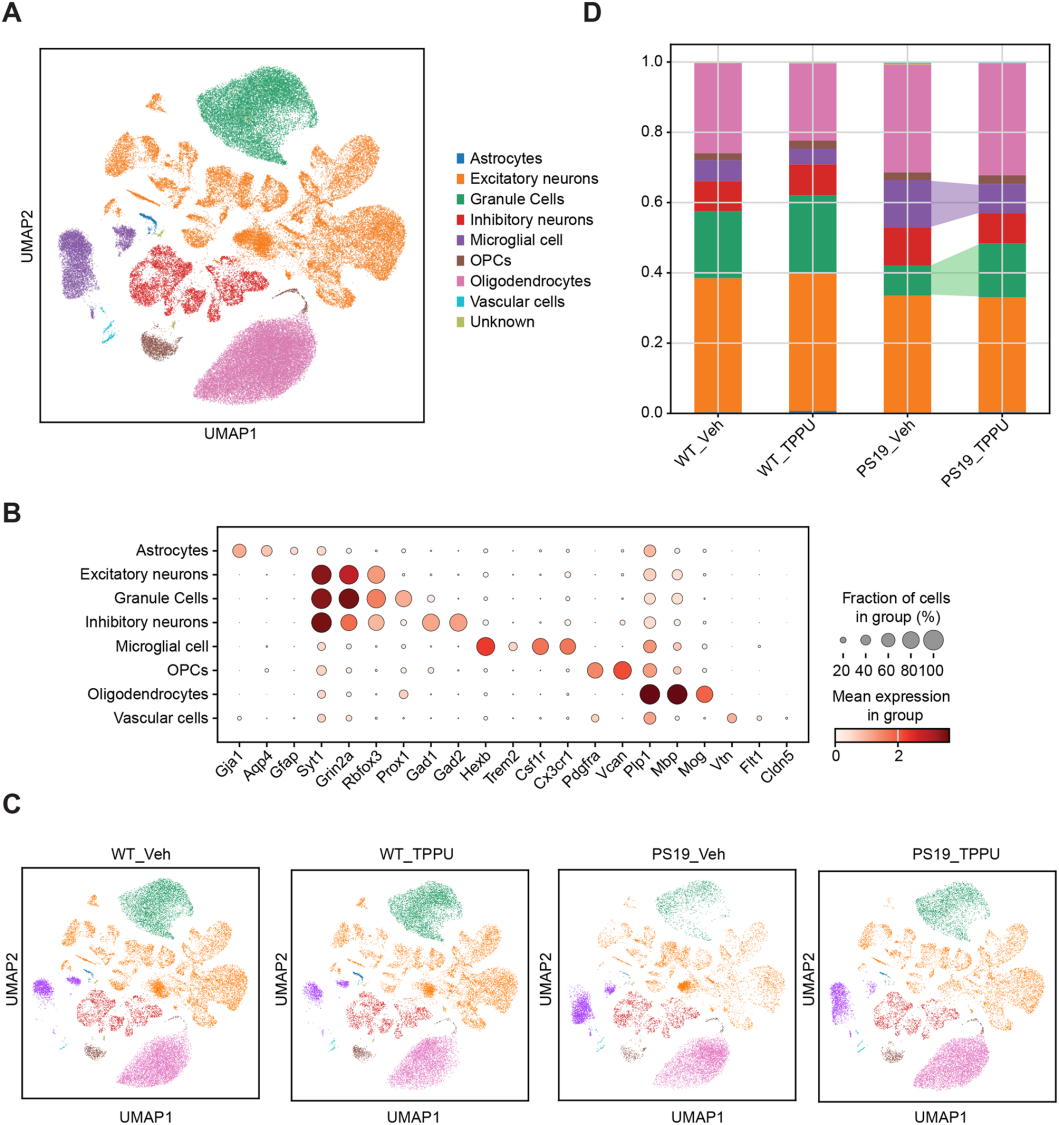
Cell type composition analysis of wild-type and PS19 mice treated with vehicle or TPPU. **(A)** UMAP embedding of snRNA-seq data showing 84,226 cells from the hippocampi of 9.5-month-old WT_Veh, WT_TPPU, PS19_Veh and PS19_TPPU mice annotated by cell types. **(B)** Dot plot showing the expression of well-known maker genes for different cell types. **(C)** UMAP embedding of snRNA-seq data across different groups. **(D)** Stacked barplot showing cell type compositions with differences in DG and MG populations between PS19_Veh and PS19_TPPU highlighted. The bars are colored by their corresponding cell classes as labeled in (A). See also Figure S5

### Mice

The PS19 mice were obtained from the Jackson Laboratory (Bar Harbor, ME). *Ephx2* null mice were provided by B. Hammock [26]. They were housed in a pathogen free mouse facility with ad libitum access to food and water on a 12 h light/dark cycle. Male and female mice were used for all experiments. All procedures were performed in accordance with NIH guidelines and approval of the Baylor College of Medicine Institutional Animal Care and Use Committee (IACUC).

### Behavioral analysis

The sEH inhibitor TPPU was synthesized as described [11], and dissolved in 10% v/v aqueous polyethylene glycol 400 (PEG400, ThermoFisher). Wild-type and PS19 mice were treated with either vehicle (1% PEG400) or TPPU at 3 mg/kg via drinking water starting at 6-6.5 months and continuously for a total of 12 weeks. Every 2 weeks, mice were supplied with a new water bottle containing fresh TPPU or vehicle.

**Fig. 4:**
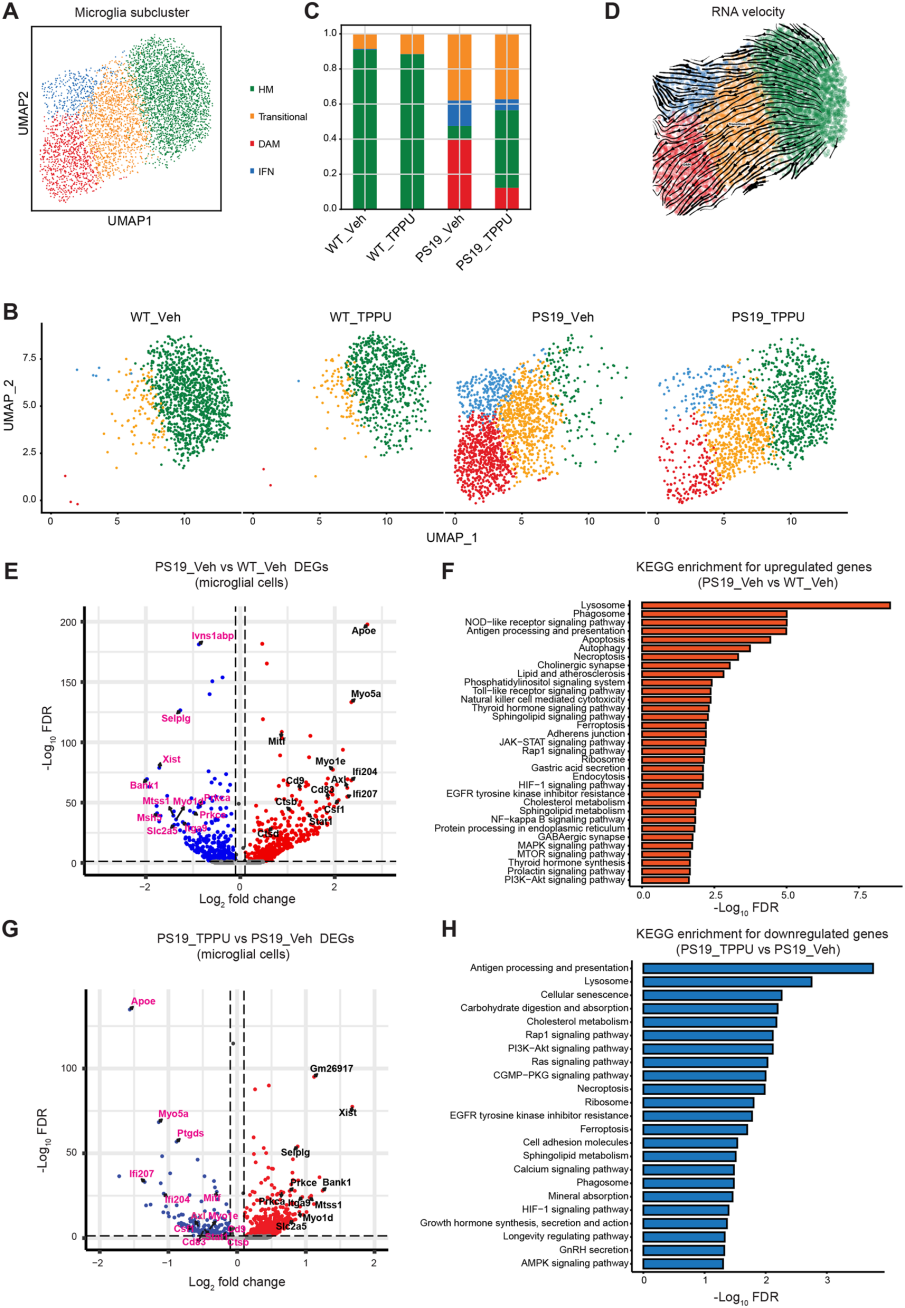
TPPU treatment suppresses DAM and IFN microglia clusters. **(A)** UMAP representation of snRNA-seq data showing 5,169 microglial cells from the hippocampi of WT_Veh, WT_TPPU, PS19_Veh and PS19_TPPU mice. HM: homeostatic microglia; DAM: disease-associated microglia; IFN: interferon-responsive microglia. **(B)** UMAP representation of snRNA-seq in WT_Veh, WT_TPPU, PS19_Veh and PS19_TPPU groups. **(C)** Stacked barplot showing compositions of microglial subclusters across different groups. The bars are colored by their corresponding cell classes as labeled in (A). **(D)** Close-up of the UMAP embedding of microglial subtypes. Arrows indicate overlaid RNA velocity streamlines showing the cell-state transitions inferred by scVelo’s dynamical mode from the displayed cells. **(E)** Volcano plot showing the differentially expressed genes in microglia of PS19_Veh relative to WT_Veh mice. Upregulated genes are highlighted in red color. Downregulated genes are highlighted in blue color. **(F)** KEGG pathway enrichment for the upregulated genes in microglial cells of PS19_Veh relative to WT_Veh. The enriched pathways are highlighted in red color. **(G)** Volcano plot showing the differentially expressed genes in microglial cells of PS19_TPPU relative to PS19_Veh mice. Upregulated genes are highlighted in red color. Downregulated genes are highlighted in blue color. (H) KEGG pathway enrichment for downregulated genes in microglia of PS19_TPPU relative to PS19_Veh. The enriched pathways for downregulated genes are highlighted in blue color. See also Figure S6

Behavioral assays were performed blind to genotype and treatment. Prior to each assay, mice were habituated in the test room (150 lx, 60 dB white noise) for at least 30 min. At least one day was given between different assays for mice to recover.

Novel object recognition was performed in a Plexiglass arene (22 cm by 44 cm). In the training day, mice were allowed to freely explore the arena with two identical LEGO objects for 10 min. Twenty-four hours post training, the mice underwent testing, in which the mice were placed in the same arena with one object previously explored during the training phase and one novel object differing in color and shape but sharing a common size and volume. The animals were allowed to freely explore the objects for 10 min. The time spent exploring each object, defined by the less than or equal to 2 cm distance between the mouse nose and the object, was measured by ANYmaze software. The exploration preference was calculated by the percentage time spent on the novel object.

**Fig. 5:**
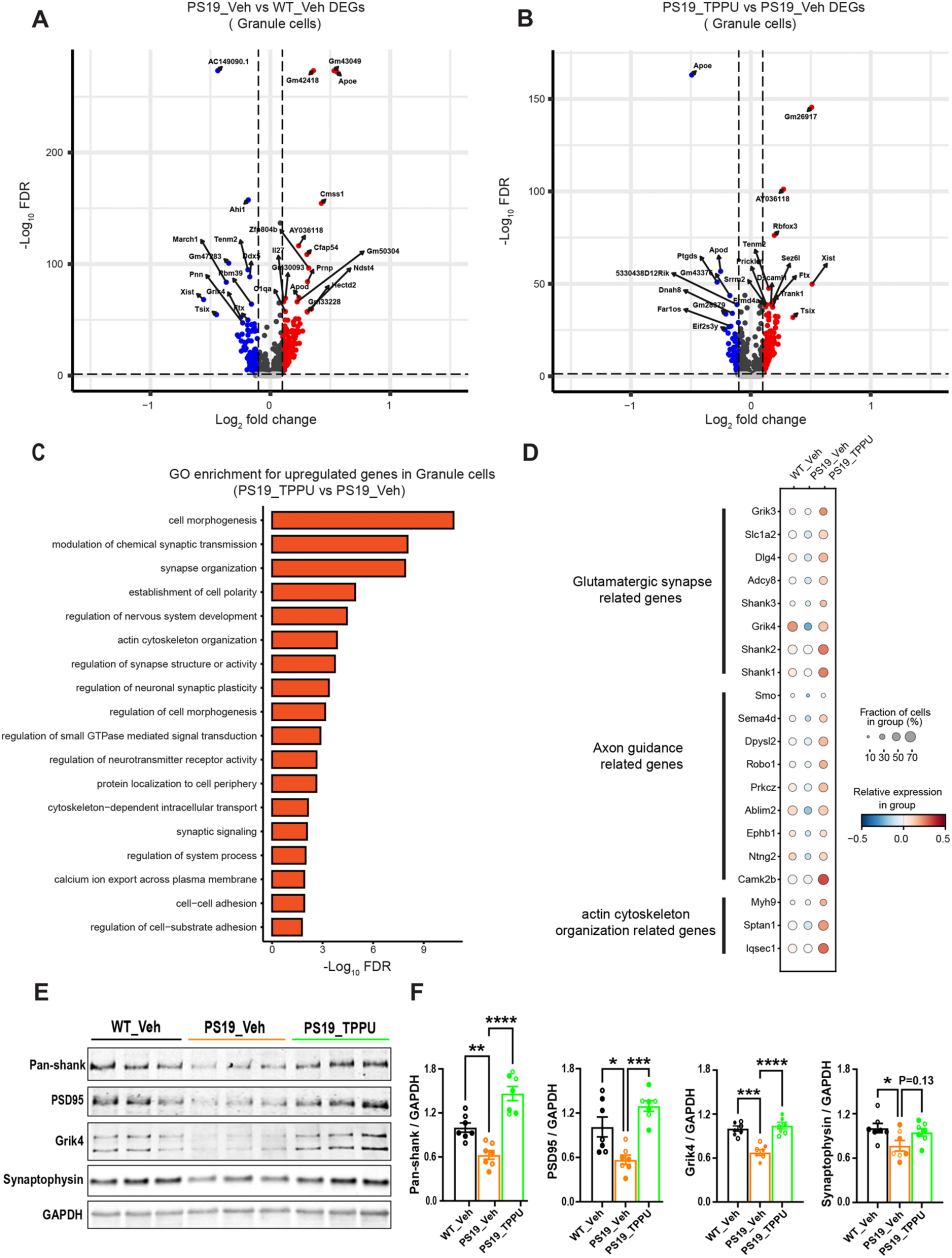
Upregulated neuronal and synaptic pathway genes in TPPU treated PS19 mice. **A** & **B**. Volcano plots showing the differentially expressed genes in granule cells of PS19_Veh relative to WT_Veh mice (A) or PS19_TPPU relative to PS19_Veh mice (B). Upregulated genes are highlighted in red color. Downregulated genes are highlighted in blue color. **C.** GO term pathway enrichment for upregulated genes in granule cells of PS19_TPPU relative to PS19_Veh mice. **D.** Dot plot showing the expression of glutamatergic synapse and axon guidance related genes that are differentially expressed in PS19_Veh and PS19_TPPU relative to WT_Veh mice (false discovery rate (FDR)-adjusted *P* < 0.01; absolute value of the log 2 (fold change) (abs. log2 (FC)) > 0.1). Dot color represents row z-scored genotype average of log- and size-normalized gene counts (normalized expression). Dot size represents the percentage of cells in a genotype with positive expression of the gene. **E.** Western blot analysis of selected pre and postsynaptic proteins in WT_Veh, PS19_Veh and PS19_TPPU hippocampal tissues. GAPDH was used as a loading control. **F.** Quantification of (E); *n* = 4♂ and 3♀ per group. Data are presented as mean ± SEM. One-way ANOVA with Tukey’s multiple comparison test. *p < 0.05; **p < 0.01; ***p < 0.001; ****p < 0.0001

The fear conditioning protocol involved a training phase, a contextual test, and a cued test as previously described [29]. During the training phase, the mice were placed in the training chamber and allowed to freely explore the environment for 2 min, then an 80-dB and 5 kHz tone was presented (auditory conditioned stimulus (CS)) for 30 s, immediately followed by a foot shock (0.8 mA) for 2 s, with the conditioning pattern repeated once. The animals were returned to their original housing cages afterwards. The contextual test was performed 24 h later by returning the mice to the same training chamber for 5 min with no presentations of shock or CS. One hour later, the cued fear test was performed by placing the mice to a cued chamber consisting of a different geometric shape, flooring, light brightness and scent. After 3 min in the chamber, the auditory stimulus was presented for 3 min. The percentage of time freezing in each trial was recorded by FreezeFrame4 (Actimetrics, Coulbourn Instruments) software.

After behavioral assays and a total of 12 weeks of treatment with TPPU or vehicle, mice were sacrificed. The brains were perfused with 0.01 M PBS (pH 7.4), microdissected, and frozen or fixed in 4% paraformaldehyde (PFA) for further analysis.

**Fig. 6:**
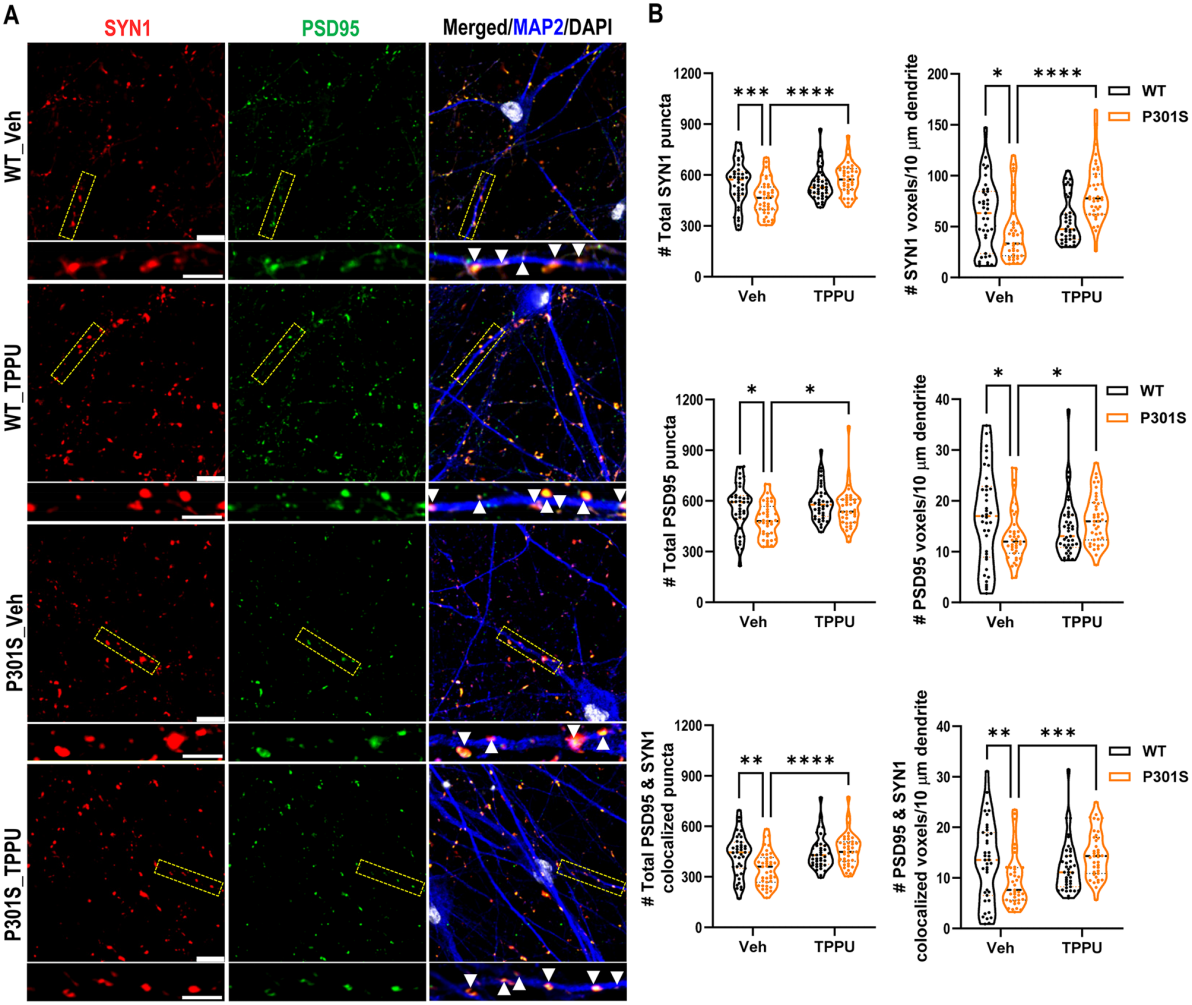
sEH inhibition enhances synaptic properties in P301S i^3^Neurons (i^3^Ns). **A**. Representative images of triple immunostaining of seven-week-old wild-type (WT) and P301S neurons treated with vehicle or TPPU (2.5 µM) using anti-synapsin1 (SYN1), anti-PSD95, and anti-MAP2 antibodies. Scale bar: 10 μm. The images beneath each main panel are enlarged views of the bracketed areas, marked with arrows indicating Syn1 and PSD95 co-localized synapses over the MAP2^+^ dendrites. Scale bar: 6 μm. **B.** Quantification of total SYN1, PSD95, and co-localized puncta, as well as the number of respective voxels aligned over 10 μm of MAP2^+^ dendrites; *n* = 39 per group. Two-way ANOVA. *p < 0.05; **p < 0.01; ***p < 0.001; ****p < 0.0001. Data are presented as mean ± SEM

**Fig. 7:**
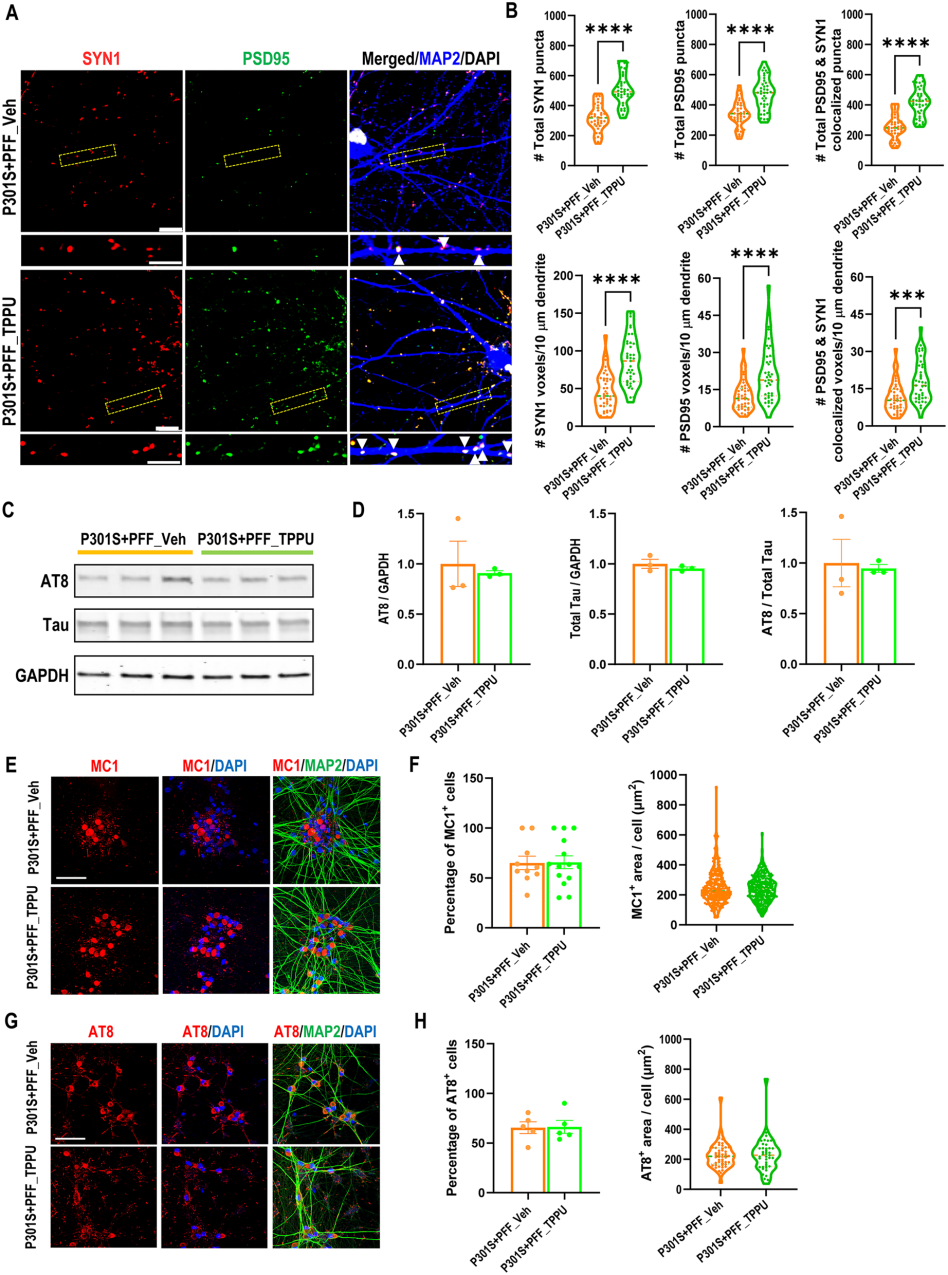
TPPU treatment improves synaptic density but not Tau pathology in PFF seeded P301S i^3^N neurons. **A**. Representative images of triple immunostaining of seven-week-old PFF seeded P301S neurons treated with vehicle or TPPU (2.5 µM) using anti-synapsin1 (SYN1), anti-PSD95, and anti-MAP2 antibodies Scale bar: 10 μm. The images beneath each main panel are enlarged views of the bracketed areas, marked with arrows indicating Syn1 and PSD95 co-localized synapses over the MAP2^+^ dendrites. Scale bar: 5 μm. **(B)** Quantification of total SYN1, PSD95, and co-localized puncta, as well as the number of respective voxels aligned over 5 μm of MAP2^+^ dendrites; *n* = 39 per group. Welch’s test. **(C)** Representative Western blots of AT8-positive phospho and total Tau in PFF-seeded P301S i^3^Ns treated with either vehicle or TPPU. GAPDH was used as a loading control. **D**: Quantification of (C); *n* = 3 per group. **E**: Representative MC1 immunofluorescence staining images of PFF-seeded P301S i^3^Ns treated with either vehicle or TPPU. Scale bar: 50 µm. **F**: Quantification of percentage of MC1^+^ cells and the surface area of MC1^+^ Tau. P301S + PFF_Veh: 10 images; P301S + PFF_TPPU: 14 images. **G**: Representative AT8 immunofluorescence staining images of PFF-seeded P301S i^3^Ns treated with either vehicle. Scale bar: 50 µm. **H**: Quantification of percentage of AT8^+^ cells and the surface area of AT8^+^ Tau (10 images per group). Data are presented as mean ± SEM. Student’s *t* test. ***p < 0.001; ****p < 0.0001

**Fig. 8:**
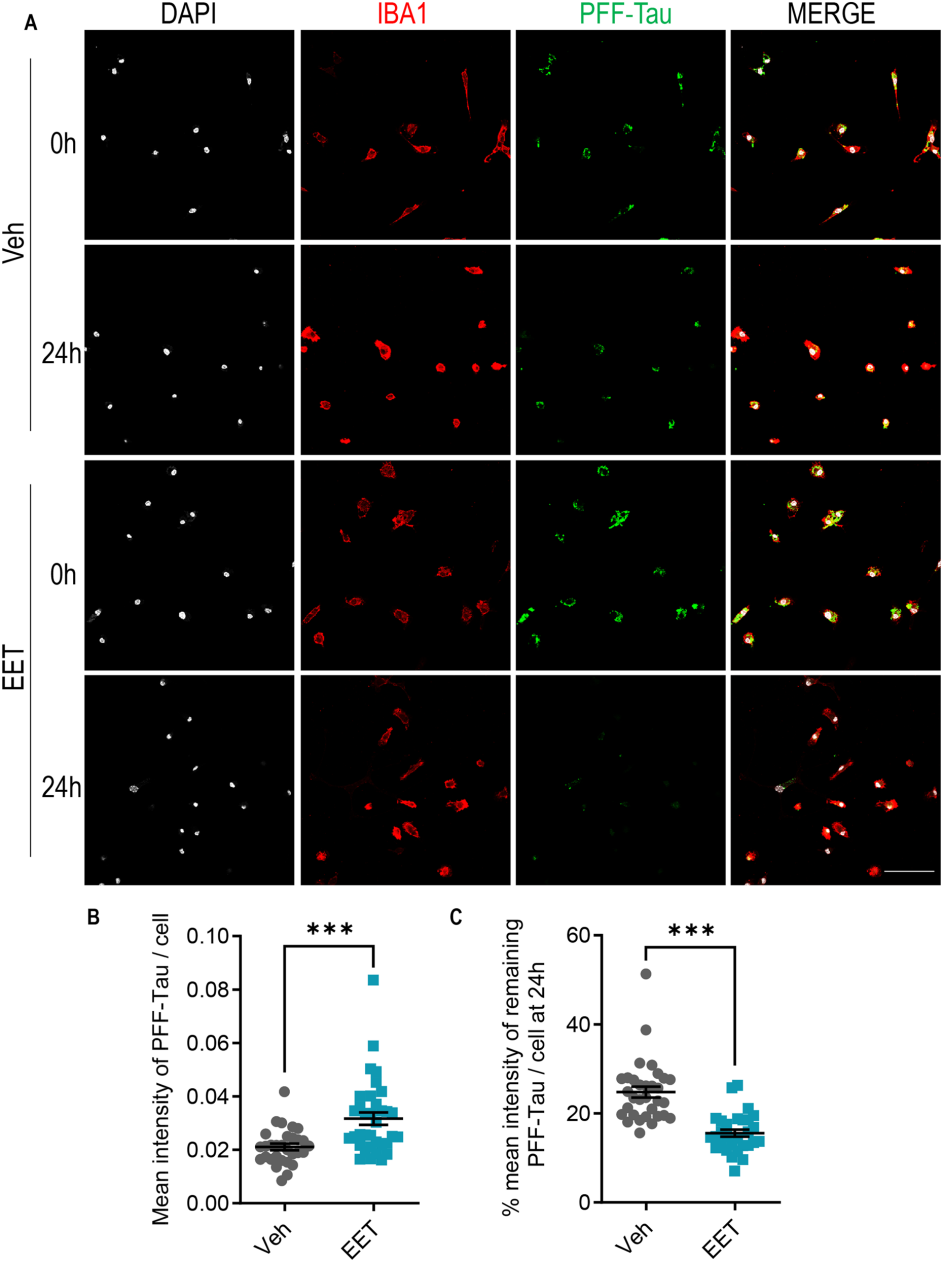
EET treatment promotes microglial phagocytosis and clearance. **(A)** Representative images of fluorescent Tau PFF (green) in Iba1 (red) positive primary microglia cultures treated with vehicle or 10 µM EET for 18 h, incubated with PFF for 6 h and imaged immediately (0 h) or after 24 h. Scale bar: 50 µm. **(B)** Quantification of mean intensity of PFF-Tau per cell at 0 h. Veh: *n* = 30 images; EET: *n* = 35 images. **(C)** Quantification of relative mean intensity of PFF-Tau per cell at 24 h. Veh: *n* = 32 images; EET: *n* = 29 images. Data are presented as mean ± SEM. ***p < 0.001 by Student’s *t* test

### Unbiased stereology

Unbiased stereology was performed in brain slices from 9.5-10-month-old mice as previously described [30]. Briefly, brain sections were immunofluorescence stained using an anti-NeuN antibody. The number of neurons in selected region was counted from six slides of sagittal Sect. (30 μm thickness, 300 μm apart). Unbiased stereology was performed using optical dissector method on a Zeiss microscope with motorized stage. Data acquisition and analysis was performed using Stereo Investigator software by MBF Bioscience. The selected region of counting was outlined under 10× objective lens, NeuN-positive neurons were counted in counting frame area of 2500 µm^2^ with sampling grid area of 4900 µm^2^ under 40× objective lens and 15 optical counting dissectors.

### Single nucleus RNA-sequencing and data analysis

The snRNA-seq was performed as described [31]. Specifically, the 9.5-month-old WT_Veh, WT_TPPU, PS19_Veh and PS19_TPPU mice (2 M + 2F/group) were perfused transcardially with cold saline. Brain hippocampi were dissected and dissociated, nuclei were stained by Hoechst-33342 and collected via the SONY SH800 FACS sorter. For each 10x Genomics run, 100k–400k nuclei were collected. 16k nuclei for each channel were loaded into the 10x controller. snRNA-seq library was performed by the 10x Genomics 3’ v3.1 kits. All PCR reactions were performed using the Biorad C1000 Touch Thermal cycler with 96-Deep Well Reaction Module. 13 cycles were used for cDNA amplification and 16 cycles were used for sample index PCR. As per 10x protocol, 1:10 dilutions of amplified cDNA and final libraries were evaluated on a bioanalyzer. The male and female samples were pooled sequenced using 100 cycle run kits and the Single Index configuration. Gene expression libraries were sequenced using paired end 150 bp reads to an average 60–70% sequencing saturation on an Illumina Novaseq.

Raw sequencing files were demultiplexed and gene expression libraries were processed using bcl2fastq. After mapping sequencing reads to mm10 reference genome by cellranger-7.1.0, the ambient RNA for each single cell was estimated and removed by Cellbender [32]. Next, doublet cells were identified using Scrublet from the filtered feature barcode matrices produced by Cellbender [33]. Cells were scored as candidate doublets by Scrublet and removed if their doublet score exceeded 0.25. Finally, remaining cells were filtered to have less than 5% of their UMI’s mapped to mitochondrial genes and to express greater than 200 genes by Scanpy [34].

Single-cell analysis was conducted by following the Scanpy pipeline (v1.9.5). Single cells were batch corrected and projected into a low-dimensional representation by single cell Variational Inference [35]. Major cell types and subclusters were marked and identified using well-known marker genes. Spliced and unspliced transcript counts were determined using the run10x command from velocyto and normalized and smoothed across 15 nearest neighbors using the filter_and_normalize and moments functions from the scVelo [36, 37]. Global scVelo velocity, transition matrix estimates and streamline visualizations were then calculated using the velocity and velocity_embedding_stream functions from the scVelo package with Dynamical Modeling. The normalized spliced and unspliced transcript counts act as the input matrix for the pseudo-time trajectory inference and the visualization of pseudotime UMAP. Differential gene expression analysis was performed between different groups and subtypes by using the Wilcoxon test implemented in the “FindMarkers” function, only genes that passed FDR < 0.05, average log2 fold change > 0.1, and % non-zero expressing cells > 10% were retained as differentially expressed genes.

### Tau preformed fibrils (PFF) Preparation

Human truncated tau containing four microtubule-binding repeats (4R) with a 5′ Myc tag was cloned into the pRK172 plasmid (a generous gift from Virginia Lee) and expressed in BL21(DE3) cells. Recombinant tau was purified as described previously [38] with minor modifications. Briefly, protein expression was induced with 1 mM IPTG for 2 h. The cell pellets were resuspended on ice in 40 mL of buffer containing 750 mM NaCl, 20 mM NaF, 100 mM MES (adjusted to pH 7.0 with 5 N KOH), 10 mM EGTA, and 5 mM MgSO4, supplemented with protease inhibitors (Recom ProteaseArrest™ Protease Inhibitor Cocktail; G-Biosciences), PMSF (0.1 mM), and lysozyme (1 mg/mL). After incubation for 20 min, DTT (5 mM) and 10% Triton X-100 (4 mL) were added. The mixture was then boiled in water for 20 min with loosely capped tubes and subsequently cooled on ice for 10 min. After ultracentrifugation at 186,000 × g for 30 min at 4 °C, the supernatant was collected and subjected to dialysis against FPLC buffer (20 mM MES, pH 6.8, 10 mM NaCl, 1 mM EGTA, 1 mM MgSO4, 5 mM DTT), supplemented with 0.1 mM PMSF. The dialyzed sample was then purified using ion exchange chromatography on a 5 mL GE HiTrap SP HP column. Fractions containing tau were identified by Coomassie-stained SDS-PAGE and further purified using a Pierce High-Capacity Endotoxin Removal Spin Column (ThermoFisher). The protein was concentrated using an Amicon Ultra-15 centrifugal filter (3-kDa MWCO) and buffer-exchanged to PBS. The protein concentration was determined using a BCA assay, and tau monomers were snap-frozen and stored at − 80 °C.

Tau fibrillization was initiated by incubating tau monomer (120 µM in PBS) with 10 µM DTT, 40 µM heparin, and 0.1% sodium azide in a shaker at 37 °C and 200 rpm for 3 days. Fibrils were pelleted by ultracentrifugation at 130,000 rpm at 4 °C and resuspended in PBS. The fibril suspension was sonicated using a probe sonicator at 40% amplitude (30 s on/off cycles for 10 min). The concentration of PFF was determined using a BCA assay.

For fluorescent labeling, 170 µL of tau fibrils (100 µM) were mixed with 1 µL of Alexa Fluor 488 (10 mg/mL), 20 µL of 1 M sodium bicarbonate, and 9 µL of PBS. The reaction was incubated at room temperature in the dark for 1 h. After labeling, the solution was centrifuged to pellet large aggregates. The supernatant, containing smaller fibrils, was passed through Zeba™ Spin Desalting Columns (7 K MWCO; ThermoFisher) to remove excess dye. The pellet was washed twice with PBS to eliminate unbound dye. Finally, the washed pellet was combined with the column-treated supernatant and sonicated to produce smaller, uniform PFFs for downstream applications.

### Human iPSC-derived neuronal cultures and treatments

The method for culturing isogenic human WT and P301S 4R-tau iPSC lines and differentiation to i^3^N neurons were adapted from [27]. Specifically, cells were maintained in Matrigel-coated 6 cm petri dishes (Millipore Sigma) using mTeSR Plus medium and passaged with Accutase (Stem Cell Technologies). For pre-differentiation, iPSCs were plated at 1.5 × 10^6^ cells per well in Matrigel-coated 6-well plates (Millipore Sigma) and cultured in Knockout DMEM/F-12 medium (ThermoFisher) supplemented with 2 µg/mL doxycycline (Sigma-Aldrich), 1X N2 supplement (ThermoFisher), 1X Non-essential amino acids (ThermoFisher), 10 ng/mL BDNF (PeproTech), 10 ng/mL neurotrophin-3 (PeproTech), 1 µg/mL mouse laminin (Millipore Sigma), and 2 µM ROCK inhibitor (Tocris). The medium was replaced 24 h later, omitting ROCK inhibitor, and pre-differentiation was maintained for 3 days (day − 3 to day 0). On day 0, pre-differentiated precursor cells were dissociated with Accutase and re-plated onto poly-D-lysine (PDL) and laminin-coated coverslips at a density of 5 × 10^4^ cells per coverslip in 24-well plates. Neuronal cultures were established in a 1:1 mixture of Neurobasal-A (ThermoFisher) and DMEM/F-12 media, supplemented with B27 and N2 (ThermoFisher), 0.5X GlutaMAX (ThermoFisher), 1X non-essential amino acids, 10 ng/mL BDNF, 10 ng/mL NT-3, 1 µg/mL mouse laminin, and 2 µg/mL doxycycline. On day 7, doxycycline was removed, and half of the medium was replaced every other day up to a total of 6 additional weeks (day 49). Cells were treated with either vehicle (DMSO) or TPPU (2.5 µM) along with the half medium change. The half of the medium was replaced every other day with fresh i^3^N maturation medium containing the respective treatments (vehicle or TPPU) to maintain a final concentration of TPPU at 2.5 µM until the cells were collected. Tau PFF was added to P301S i^3^Ns at 1.5 mg/mL final concentration once on day 7, either with vehicle (DMSO) or TPPU (2.5 µM). Subsequent medium changes were performed every other day with fresh i^3^N maturation medium containing only the respective treatments (vehicle or TPPU) to maintain a final concentration of TPPU at 2.5 µM until the cells were collected.

### Primary microglia cultures and treatment

Primary microglia cultures were prepared as described previously [39]. In brief, mouse cortices and hippocampi were isolated from postnatal day 1 to day 3 pups. Tissue was digested with 2.5% trypsin at 37 °C for 15 min, centrifuged, triturated, and resuspended in DMEM medium with 10% FBS. Cells were plated onto PDL–coated T-75 flasks to generate mixed glial cultures. These mixed glia cultures were allowed to grow for 2 weeks, with refeeding every other day. The mixed glia cultures were subjected to separation via CD11b microbeads selection according to the manufacturer’s instructions (Miltenyi Biotec). Enriched microglia were plated in 24 well PDL-coated plates in DMEM with 10% FBS and 1% Pen/Strep and supplemented with 10 ng/mL M-CSF. Microglial cells were allowed to rest for 24 to 48 h before treatments.

The cells were treated with vehicle (DMSO) or 11,12- EET (10 µM) for the next 18 h. For Tau fibril phagocytosis assay, fluorescently labelled (Alexa-488) Tau fibrils at 2.5 µg/mL were added to the culture for an additional 6 h. The Tau containing media was removed and replaced with 0.01% Trypsin for 5 min to eliminate Tau attached to the cell surface. Thereafter the cells were washed with PBS. One set (0 h) was fixed in 4% PFA for Tau phagocytosis, the other set was incubated in fresh media for another 24 h to measure Tau clearance. Images were acquired by the Leica STELLAS confocal microscope using 40X objective.

### Western blotting

Cells or brain tissues were collected and resuspended in modified radioimmunoprecipitation (RIPA) assay buffer containing protease and phosphatase inhibitor mixture. Cell suspensions were sonicated after resuspension, whereas mouse brain tissues were homogenized, sonicated, and then centrifuged at 14,000 x g for 45 min at 4^°^C, as described previously [23]. Protein concentrations were estimated using a BCA kit (Thermo Fisher). Lysates were separated on 7.5–15% SDS-polyacrylamide electrophoresis gels (Bio-Rad). After the separation, proteins were transferred to a nitrocellulose membrane, and nonspecific binding sites were blocked by treating with either Odyssey blocking buffer (LI-COR) or TBS with 5% bovine serum albumin (BSA) followed by antibody incubation. The bands were quantified using Fiji ImageJ software and normalized to the loading control. The primary antibodies used are described in Table S3.

### Immunofluorescence staining

Mice were anesthetized and perfused transcardially with PBS and fixed in 4% PFA overnight at 4 °C, dehydrated with 30% sucrose in PBS and serially sectioned at 30 μm on a sliding microtome (Leica). Free floating sections were blocked with 10% donkey serum and 3% BSA in PBS with 0.3% Triton X-100 for 1 h at room temperature. Sections were then incubated with primary antibodies (see Table S3 for details) in blocking solution overnight at 4 °C. On the next day, sections were washed and incubated with secondary antibodies and DAPI in blocking buffer for 1 h at room temperature. After washing with 0.1% Triton X-100 in PBS, sections were mounted with ProLong Gold anti-fade reagent (Invitrogen).

Primary cultures or i^3^Ns grown on coverslips were fixed in 4% PFA for 20 min, permeabilized with 0.3% Triton X-100 in PBS for 5 min at room temperature. Coverslips were then incubated in blocking buffer (5% normal donkey serum, 0.01% Triton X-100 in PBS) for 1 h at room temperature. After blocking, coverslips were incubated with primary antibodies (see Table S3 for details) overnight in blocking buffer at 4 °C. Coverslips were then washed in PBS followed by incubation with secondary antibodies and DAPI in blocking buffer for 1–1.5 h at room temperature. After brief washing with PBS, coverslips were then mounted on slide glasses with ProLong Gold antifade reagent.

### Image quantification

Area fluorescence in the hippocampus was averaged across at least 4 consistently represented Sect. (300 μm apart). Images were obtained with a Leica confocal microscope and analyzed by Fiji ImageJ. Background fluorescence was subtracted by the software before quantification.

In vivo synaptic colocalization quantification was performed as previously described [40]. Images were captured using a 63X oil lens with 6X digital zoom. Z-stacks of 10 μm thickness were obtained with a 0.2 μm step size. Z-stacks with pre- and postsynaptic puncta were analyzed using the Spots feature of IMARIS. Spots were generated automatically with manual adjustment for accurate puncta representation for each channel separately, and total number of spots were recorded for each channel, and analyzed by the Co-localize Spots MATLAB (MathWorks) plugin. Pre- and postsynaptic puncta were defined as colocalized if their centers were within 200 nm.

For in vitro synaptic colocalization quantification, images were captured using a 63X oil lens with 2.5X digital zoom. Synapses were identified as puncta positive for the presynaptic marker SYN1 and postsynaptic marker PSD95, in proximity to the dendritic marker MAP2. Dendritic length and the number of synaptic puncta were measured using IMARIS software (Bitplane), employing the filament feature for MAP2^+^ dendrites and the spot feature for SYN1 and PSD95 puncta. Colocalization was quantified by counting the number of PSD95 puncta colocalized with SYN1 puncta within a 1 μm distance [41]. Synaptic density was determined by using the Coloc-channel in IMARIS software and calculating the number of voxels or volumetric pixels in SYN1, PSD95, or their colocalized puncta divided by the length of the dendrite.

For microglia morphology quantification, Iba1-positive microglia were imaged by confocal microscopy using a 63X oil lens with 4X digital zoom to generate Z-stacks of the tissue thickness (~ 30 μm) with a step-size of 0.2 μm. Images were analyzed using IMARIS software, the filament function was used to generate filaments for individual cell in the images and microglia processes were automatically rendered based on the Iba1 signal.

### Statistical analysis

All data were analyzed with GraphPad Prism 10.4.1 and presented as mean ± SEM (*p < 0.05, **p < 0.01, ***p < 0.001 and ****p < 0.0001). For simple comparisons, Welch’s or student’s *t*-test were used. For multiple comparisons, one- or two-way ANOVA with Tukey’s multiple comparison test were used. All samples or animals were included in the statistical analysis unless otherwise specified.

## Results

### sEH inhibition rescues neuronal and synapse loss and cognitive impairment in PS19 mice

We reported that sEH levels were elevated Aβ mouse models and AD brain samples [23]. To assess whether it is also the case in tauopathy diseases, we performed Western blot analysis of total and phospho-Tau levels in the hippocampus of PS19 transgenic mice and medial frontal cortices of human tauopathy patients including corticobasal degeneration (CBD), Picks disease, and progressive supranuclear palsy (PSP) (Supplementary Figure S1). While the sEH levels were comparable between WT and PS19 mice at 6 months, it was significantly higher in PS19 mice at 9 months when frank Tau pathology develops (Fig. S1, A-D). Upregulation of sEH expression was also observed in postmortem tauopathy patient samples compared to non-cognitively impaired controls (Figures S1E and S1F). These results implicate a dysregulation of the sEH pathway by Tau pathology and provide the rationale for testing the therapeutic effect of sEH inhibition in tauopathy conditions.

Accordingly, we administered the sEH inhibitor TPPU to PS19 mice of both sexes at 3 mg/kg via drinking water starting at 6-6.5 months when neuropathological changes start to manifest and continuously for 3 months [23]. Vehicle or TPPU-treated littermate wild-type (WT) mice were used as controls. We chose 9.5–10 months for analysis because Tau pathology, neuronal loss, immune system changes and behavioral deficits can be readily detected in PS19 animals [28]. Measurement of water consumption and body weight of WT and PS19 mice showed reduced body weight in male PS19_Veh group compared to WT_Veh, but no statistically significant differences between vehicle and TPPU treated groups (Figure S2), indicating that TPPU does not exert adverse reactions. Likewise, open field test revealed no group differences in locomotor activities or movement behavior (Figure S3, A-D).

We next evaluated the effect of TPPU in cognition using the novel object recognition (NOR) test (Fig. 1A), which assesses the hippocampus dependent long-term recognition memory by measuring the percent time spent exploring a novel object. The four groups did not exhibit object bias during the training phase (Figure S3E). However, compared with the vehicle treated WT (WT_Veh) mice, the PS19_Veh group displayed no preference to the novel object (Fig. 1A). TPPU treatment did not exert any appreciable effect in WT mice (WT_TPPU) similar to our previous study [23], but significantly improved the NOR score in PS19 mice (PS19_TPPU, Fig. 1A). We further performed fear conditioning to test the hippocampal dependent (contextual) and independent (cued) associative learning (Fig. 1B). All groups showed similar freezing percentage during the conditioning phase (Figure S3F). In the context test, the PS19_Veh group exhibited significantly decreased freezing percentage compared to WT_Veh or WT_TPPU groups, suggesting an impaired contextual memory. TPPU treatment of PS19 mice resulted in a close to significant increase (*p* = 0.071) in freezing frequency compared to the PS19_Veh group. The percentage of freezing in the post-cue test was indistinguishable between groups. Thus, TPPU treatment mitigated hippocampus-dependent memory impairment in PS19 mice.

To investigate the underlying mechanism of improved behavior by TPPU, we measured the NeuN-positive neurons in hippocampal area CA1 and dentate gyrus (DG) using unbiased stereology [30]. The results showed substantially reduced numbers in PS19_Veh group compared to WT_Veh and WT_TPPU controls and partial but significant rescue by TPPU treatment (Fig. 1C and D). Similar results were obtained by co-immunostaining of pre- and post-synaptic proteins Bassoon and Homer1, respectively, and quantifying the number of pre- and post-synaptic as well as co-localized puncta (Fig. 1E and F). Overall, these results demonstrate neuroprotective and synaptic promoting properties of TPPU, providing basis for its rescue of behavioral deficits in PS19 mice.

### TPPU treatment ameliorates Tau pathology and neuroinflammation in PS19 mice

Having demonstrated a beneficial role of TPPU in reversing neuronal phenotypes of PS19 mice, we asked whether sEH inhibition could influence Tau pathology. Immunostaining of area CA1 of the hippocampus using AT8 and MC1 antibodies (Fig. 2A and B) and Western blotting using PHF1 and AT8 (Fig. 2C and D) both showed significant reductions in TPPU treated PS19 mice.

The development of Tau pathology is accompanied by reactive gliosis and neuroinflammation. Indeed, immunostaining using anti-Iba1, -GFAP and -COX2 antibodies showed higher microglia and astrocyte immunoreactivities and proinflammatory marker COX2 levels in PS19 mice, all of which were significantly reduced by TPPU treatment (Fig. 2E and F), consistent with the anti-inflammatory activity of sEH blockade reported before [20]. Examination of microglia morphology using IMARIS imaging documented reduced filament length and branches and increased cell volume and surface area in PS19 microglia, which were normalized by TPPU treatment (Fig. 2G and H). Reduced Tau pathology and neuroinflammation was also observed by genetic knockout of *Ephx2* on PS19 background (Figure S4) [26].

### Reversal of microglial States in TPPU treated PS19 mice

We next sought to interrogate the cell types contributing to TPPU-mediated changes in PS19 mice by conducting single-nucleus RNA-sequencing (snRNA-seq) of hippocampal samples collected from 9.5-month-old WT and PS19 mice treated with vehicle or TPPU. Nuclei were isolated by fluorescence activated cell sorting (FACS). After stringent quality control including doublet removal, batch effect correction and normalization (Figure S5), we obtained a total of 84,226 high-quality single cell transcriptomes (Supplementary Table 2), which were annotated into 8 major cell types based on the expression of well-known cell-type-specific markers (Figs. 3, A-C). Consistent with our previous study [31], cell type composition analysis revealed expanded microglia and loss of DG cells in PS19_Veh group compared to WT_Veh controls (Fig. 3D). Consistent with the immunostaining results, TPPU treatment resulted in robust reductions of microglia population and restoration of DG cells.

Further analysis of microglia population identified four subclusters: homeostatic (HM), transitional, disease-associated (DAM), and interferon-responsive (IFN) (Fig. 4A and Figure S6A). We found no appreciable differences between WT_Veh and WT_TPPU microglia, with both consisting mostly the HM subtype (Fig. 4B and C). In PS19 microglia, the HM cluster was drastically diminished while the transitional, DAM and IFN clusters were greatly expanded. Pseudotime trajectory analysis based on RNA velocity indicated that DAM was derived from transitional microglia while IFN may be directly converted from HM (Figs. 4D). TPPU treatment led to substantial reductions of DAM and IFN clusters and partial replenishment of the HM pool while the transitional microglia population remained largely the same. Pathway analysis of differentially expressed genes (DEGs) showed that, compared to HM, the DAM was enriched in innate immune responses, endocytosis, and lysosomal pathways (Figures S6C and S6D), while the anti-viral defense and cytokine stimulus pathway genes were upregulated in the IFN subtype (Figures S6E and S6F). Both the DAM (*Apoe*, *Axl*, *Cd9*, *Csf1*, *Ctsb*, *Ctsd*, and *Mitf*) and IFN (*Ifi204*, *Ifi207* and *Stat1*) genes were upregulated in PS19_Veh microglia and suppressed by TPPU treatment (Fig. 4E and G). KEGG enrichment analysis identified lysosome, antigen processing and presentation, and cholesterol and lipid metabolism as top enriched pathways upregulated in PS19_Veh microglia, which were downregulated by TPPU (Fig. 4F and H). These results demonstrate a broad reversal of microglial states by sEH inhibition.

### TPPU promotes glutamatergic synapses in a cell-autonomous manner

Transcriptomic analysis of dentate granule cells across groups revealed limited DEGs (Fig. 5A). Regardless, GO term analysis documented that, while comparison of PS19_Veh vs. WT_Veh failed to identify enriched pathways, TPPU treatment of PS19 mice resulted in the upregulation of multiple neuronal and synapse pathway genes compared to vehicle treated group (Fig. 5B). These included genes related to glutamatergic synapse function, axon guidance and actin cytoskeleton organization (Fig. 5C). Of note, numerous postsynaptic genes, including *Dlg4*, *Grik3*, *Grik4*, *Shank1*, *Shank2* and *Shank3*, were downregulated in PS19_Veh group, and were rescued by TPPU treatment (Fig. 5C). Western blot analysis of selected pre- and postsynaptic proteins from brain lysates revealed reduced synaptic protein levels in PS19_Veh group compared to WT_Veh. In line with the gene expression data, TPPU treatment of PS19 mice resulted in a significant rescue of postsynaptic (Pan-Shank, PSD95 and Grik4) proteins. Although the presynaptic protein synaptophysin was also increased, it failed to reach statistical significance (Fig. 5D and E). These results indicated a potentially preferential effect of TPPU on postsynaptic compartment.

The improved neuronal survival and function in TPPU treated PS19 mice may be mediated by a direct effect of sEH inhibition in neurons or indirectly through immune pathway modulation. To distinguish these possibilities, we differentiated human iPSCs expressing isogenic 4-repeat (4R) WT or P301S mutant Tau to excitatory neurons (i^3^Ns) [27], and treated the cultures with either DMSO (vehicle) or 2.5 µM TPPU (Fig. 6). Immunostaining of seven-week-old WT and P301S i^3^Ns with synapsin-1 (SYN1) and PSD95, alongside MAP2 to mark the dendrites, revealed a significant reduction in SYN1, PSD95 and colocalized SYN1/PSD95 puncta in P301S i^3^Ns compared to that of WT. Treatment with TPPU significantly restored the number of total SYN1, PSD95 and colocalized puncta, as well as the number of voxels per 10 μm of MAP2-positive dendrites, in P301S i^3^Ns.

We next seeded the P301S i^3^Ns with K18 pre-formed fibrils (PFF) at day 7 of differentiation to induce Tau aggregation [27, 31], and treated the cultures with vehicle or 2.5 µM TPPU continuously for a total of seven weeks. Immunostaining indicated that TPPU treatment increased both the total number and voxels of SYN1, PSD95 and colocalized puncta per 10 μm of MAP2-positive dendrites in P301S i^3^Ns seeded with PFF (Fig. 7A and B). Intriguingly, neither the total Tau nor AT8-positive Tau were affected by TPPU treatment (Fig. 7C and D). Immunostaining also showed comparable levels of MC1 and AT8 immunoreactivities (Figs. 7, E-H). Thus, sEH inhibition conferred a synaptoprotective effect in mutant Tau i^3^Ns under both aggregated and non-aggregated conditions without altering Tau pathology.

### Improved microglial phagocytosis and clearance by EET

Tau is known to be secreted from neurons and Tau pathology can transmit in a prion-like spreading manner [42–45]. As professional phagocytes, microglia actively take up and degrade extracellular materials, including Tau. Since sEH inhibition did not have an appreciable effect on Tau accumulation in iPSC-derived neurons, we wondered whether the reduced Tau pathology by TPPU treatment in PS19 mice could be due to enhanced Tau uptake and clearance by microglia. We have shown that microglia do not express sEH but addition of exogenous 11,12-EET (herein referred to as EET) can suppress microglia inflammation [23]. We thus tested whether EET may modulate Tau phagocytosis and clearance in primary microglia cultures by first treating the cultures with vehicle (DMSO) or 10 µM EET for 18 h, followed by adding fluorescently labelled Tau PFF (2.5 µg/ml) for 6 h and quantifying the intracellular fluorescence either at time 0 (phagocytosis) or 24 h later (clearance). The results showed that addition of EET effectively increased both the uptake and clearance of Tau PFF (Fig. 8). Therefore, the reduced Tau pathology by TPPU treatment in vivo may be attributed by improved microglia phagocytosis and Tau clearance through increased EETs.

## Discussion

Here we investigated the role of the sEH-EET axis in Tau pathogenesis. We demonstrate that blockade of sEH, either by pharmacological inhibition or genetic ablation, reverses heightened microglia states and rescues neuronal and synaptic loss in PS19 mice. This effect is accompanied by reduced Tau pathology and improved cognitive function. Mechanistically, we uncover a dual role of the sEH-EET pathway in neurons and microglia: sEH inhibition promotes neuronal and synaptic health in a cell-autonomous manner, while EET supplementation enhances microglial phagocytosis and clearance.

Elevated sEH levels have been reported in several neurological disorders, including depression, Parkinson’s disease and AD, and sEH inhibition has shown therapeutic benefits in preclinical models [14, 16, 23, 25, 46–48]. These protective effects are largely attributed to increased circulating EETs resulting from sEH inhibition, which act on microglia to suppress neuroinflammation. While this mechanism is relevant in tauopathy, we provide evidence that sEH plays an independent role in neurons. Specifically, our snRNA-seq revealed that multiple genes related to actin cytoskeleton organization, axon guidance and glutamatergic synapses are downregulated in DG neurons of PS19 mice. This aligns with previous findings that Tau, normally localized to axons, redistributes to dendrites and postsynaptic compartment under pathological conditions, where it impairs actin dynamics, spine density, and synaptic function via interactions with NMDAR, PSD95 and other synaptic proteins [49–51]. The restoration of actin cytoskeleton components and postsynaptic proteins by TPPU treatment supports a general neuroprotective role of sEH blockade, which may underlie the observed improvement in synaptic and cognitive function in PS19 mice.

Importantly, using iPSC-derived neurons, we show that TPPU treatment increases synaptic density in P301S neurons regardless of Tau inclusion status, indicating that sEH inhibition confers neuroprotective and synaptic promoting benefits via a cell-autonomous mechanism. However, the primary downstream targets of the sEH-EET pathway that mediate these widespread changes remain unclear. Additionally, our snRNA-seq was performed in hippocampal tissues. It remains to be determined whether similar findings occur in other brain regions implicated in cognitive function, such as the cortex.

Our results show that long-term TPPU treatment in mice reduces Tau pathology. However, TPPU does not affect Tau inclusions in cultured iPSC-derived neurons. Besides the intraneuronal pathology, Tau is known to be secreted by neurons and spreads in a prion-like manner via cell-to-cell transmission [42–45]. Microglia are known to uptake and clear extracellular Tau [52], and the enhanced microglial uptake of Tau fibrils following EET treatment supports the idea that reduced Tau pathology by TPPU in vivo may stem from increased microglial uptake and clearance. Nevertheless, we cannot exclude the possibility that TPPU directly affects Tau pathology in neurons and that the lack of effect in vivo may be due to system-specific differences or treatment paradigms.

While TPPU is a well-established sEH inhibitor, in vitro studies have also shown it inhibits p38 kinase [53]. Thus, its anti-inflammatory effect could reflect dual inhibition of both sEH and p38. However, the observation that genetic inhibition of *Ephx2* in PS19 mice produces similar results suggests that sEH inhibition is the primary mechanism of action. This is further supported by consistent findings across pharmacological and genetic studies in Aβ mouse models [23–25].

An anti-inflammatory activity of TPPU has been well-established. Our snRNA-seq analysis indicates that this is likely due to the prominent reductions of DAM and IFN microglial subtypes. Notably, *Mitf* and *Stat1*, key transcriptional regulators of the DAM and IFN subtypes respectively [54, 55], were elevated in PS19 and downregulated following TPPU treatment. This supports the idea that suppression of MITF and STAT1 pathways mediates the anti-inflammatory activities of TPPU. Additionally, the near exhaustion of homeostatic microglia in PS19 mice and its restoration following TPPU treatment suggest that sEH inhibition promotes microglial state transitions and rejuvenates chronically activated microglia induced by pathological Tau.

Our in vitro findings support a dual mechanism of the sEH-EET pathway in neurons and microglia: sEH inhibition in neurons directly promotes synaptic protein expression and synaptic density without altering Tau pathology, whereas EET supplementation in microglia enhances Tau phagocytosis and clearance. We propose that this combination leads to improved neuronal and synaptic function and reduced Tau pathology in PS19 mice. Nonetheless, besides neurons and microglia, other cell types and mechanisms may also contribute to the overall effect of sEH inhibition in vivo. For instance, sEH is expressed in the vasculature, where it modulates vascular inflammation and blood-brain barrier integrity [10, 13, 56]. Additionally, sEH is highly expressed in peripheral tissues. Hepatic sEH, in particular, has been implicated in stress and depression phenotypes in mice [57]. Directly relevant to AD, Wu et al. recently reported that hepatic *Ephx2* ablation reduces cerebral Aβ pathology and improves cognition, potentially through circulating EETs capable of crossing the blood-brain barrier [25]. Therefore, it is possible that TPPU exerts therapeutic effects not only centrally but also through actions in peripheral tissues and vasculature.

## Conclusions

Several classes of sEH inhibitors have been developed [58], and overall, they have shown favorable safety profiles in preclinical studies. Among these, TPPU is widely used as a tool compound due to its high potency, specificity, and favorable pharmacokinetics [11, 59, 60]. A TPPU derivative, EC2056, is under clinical investigation, with no adverse effects reported so far (ClinicalTrials.Org, NCT04908995) [61]. Multiple studies have demonstrated a dysregulation of the sEH metabolic pathway in AD and a beneficial role of sEH inhibition in Aβ mouse models. We report here that it also mitigates Tau pathology through coordinated modulation of neuronal and immune pathways. These findings, coupled with the availability of sEH positron emission tomography (PET) tracers for in vivo imaging [62], make sEH an attractive therapeutic target for AD, tauopathy diseases, and possibly other neurodegenerative disorders.

## Electronic supplementary material

Below is the link to the electronic supplementary material.

Supplementary Material 1

## Data Availability

Raw snRNA-seq data have been deposited at NCBI GEO with the accession number: GSE272373 (https://www.ncbi.nlm.nih.gov/geo/query/acc.cgi?acc=GSE272373; reviewer token: mnaxuuugpfujzmf) and are publicly available as of the date of publication. No original codes were generated in this study. Any additional materials or datasets used in the current study are available from the corresponding author on reasonable request.

## Abbreviations

Aβ: Amyloid beta
AD: Alzheimer’s disease
CBD: Corticobasal degeneration
CLU: Clusterin
CNS: Central nervous system
CS: Conditioned stimulus
DAM: Disease associated microglia
DAPI: 4’,6-diamidino-2-phenylindole
DEGs: Differentially expressed genes
DG: Dentate gyrus
DMSO: Dimethylsulfoxide
EETs: Epoxyeicosatrienoic acids
Ephx2: Epoxide hydrolase 2
4R: 4-repeat
GFAP: Glial fibrillary acidic protein
HM: Homeostatic microglia
Iba1: Ionized calcium-binding adaptor molecule 1
IFN: Interferon-responsive
iPSCs: Induced pluripotent stem cells
i^3^Ns: Integrated, inducible, and isogenic iPSC-derived neurons
KOPS19: *Ephx2* knock out crossed with PS19 mice
KEGG: Kyoto Encyclopedia of Genes and Genomes
NOR: Novel object recognition
PEG400: Polyethylene glycol 400
PFF: Preformed fibrils
PSP: Progressive supranuclear palsy
PS19: MAPT P301S transgenic mice
PTK2B: Protein Tyrosine Kinase 2 Beta
sEH: Soluble Epoxide Hydrolase
sEHKO: Ephx2 knock out mice
snRNA-seq: Single-nucleus RNA-sequencing
SYN1: Synapsin-1
TPPU: 1-trifluoromethoxyphenyl-3-(1-propionylpiperidin-4-yl) urea
WT: Wild-type
