# Supplementary material for: Inhibition of soluble epoxide hydrolase confers neuroprotection and restores microglial homeostasis in a tauopathy mouse model
Source: Mol Neurodegener. 2025 Apr 23;20:44. doi: 10.1186/s13024-025-00844-x (PMC12016400; doi:10.1186/s13024-025-00844-x)
Supplement: Supplementary file 1 — Supplementary Material 1 [file 13024_2025_844_MOESM1_ESM.pdf]

List of Supplemental Materials

- **Supplemental Methods**

Water consumption

Wild-type and PS19 mice were treated continuously via drinking water with either vehicle (1% PEG400) or TPPU at 3 mg/kg. Mice were housed in groups of 2-4 per cage, with each cage receiving the same amount of fluid in a water bottle. The remaining volume in each bottle was measured weekly using a graduated cylinder, and water consumption was normalized to the number of mice per cage.

Open field assay

The open field tests were performed using the Versamax system. Mice were placed in the center of a 40 × 40 × 30-cm chamber equipped with transparent walls (Accuscan) to record activity during a 30 min test period. General locomotor activity was detected automatically by sensor beams at X, Y, and Z directions. and were exported and analyzed with Versadat software. The time spent in the center of the arena and total distance travelled were measured.

Cytokine ELISA

The levels of TNFα, IL1β, and IL6 in the Hippocampus tissue lysates were determined using DuoSet ELISA Kits from R&D (DY410, DY401, DY406) according to the manufacturer's instructions.

- **Supplementary Figures (Figs. S1-S6) and Legends**

- **Supplementary Tables (Tables S1-S3)**

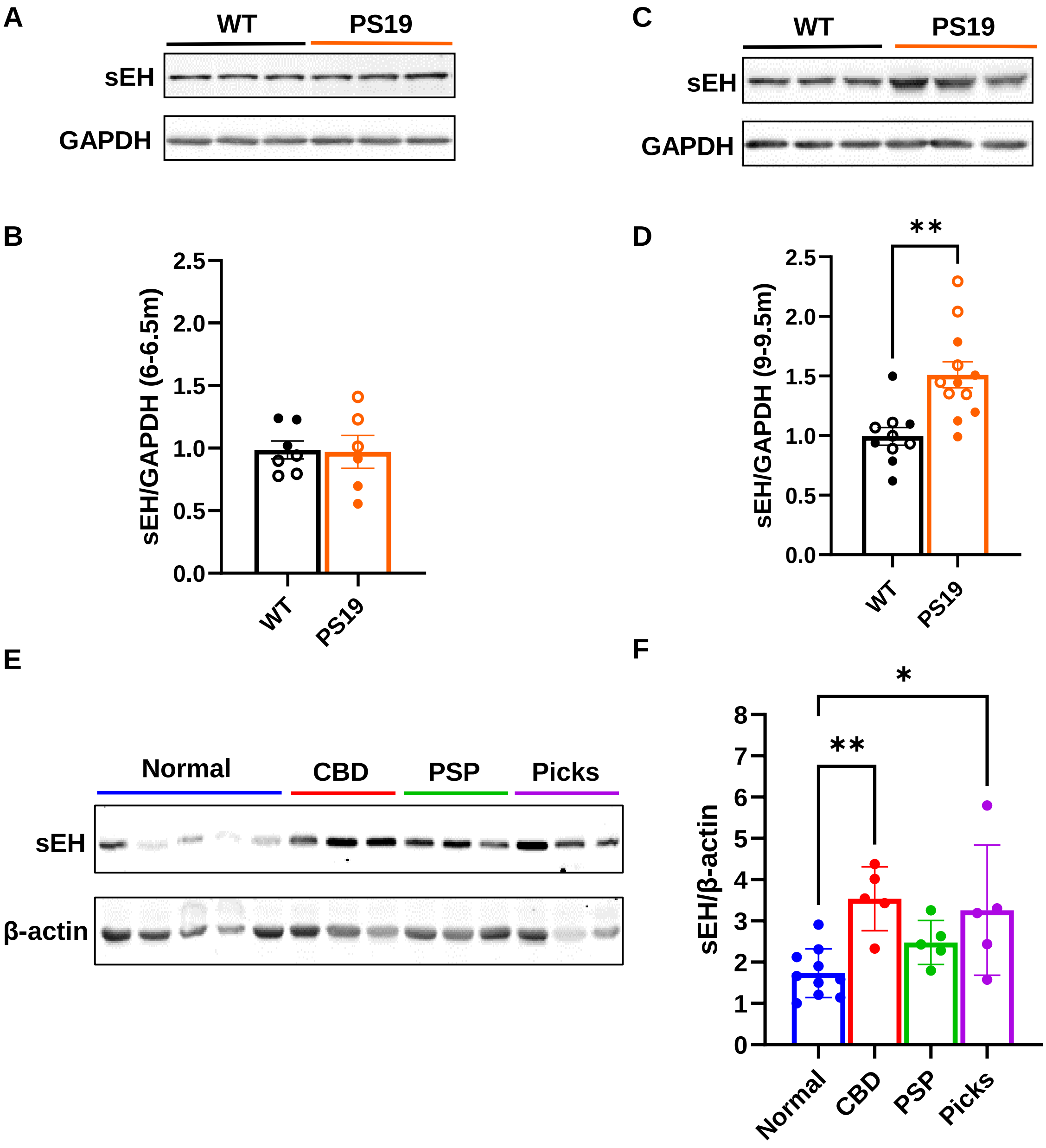

Figure S1. Increased sEH levels in tauopathy.

A & B. Representative Western blot (A) and quantification (B) of sEH levels in hippocampal samples of WT and PS19 mice at 6-6.5 months. GAPDH was used as a loading control. WT: n=3♂and 4♀; PS19: n=3♂and 3♀; Student *t*-test. **C & D.** Representative Western blot (C) and quantification (D) of sEH levels in hippocampal samples of WT and PS19 mice at 9-9.5 months. GAPDH was used as a loading control. WT: n=5♂and 5♀; PS19: n=6♂and 6♀; Student *t*-test. **E & F.** Representative Western blot (E) and quantification (F) of sEH levels in frontal cortex of non-impaired (Normal) and CBD, PSP and Picks subjects. β-actin was used as a loading control. Normal: n=10; CBD: n= 5; PSP: n=5; Pick's: n=5; one-way ANOVA with Tukey's multiple comparison test. Filled circle: male, open circle: female. Data are presented as mean ± SEM. *$p < 0.05$; **$p < 0.01$.

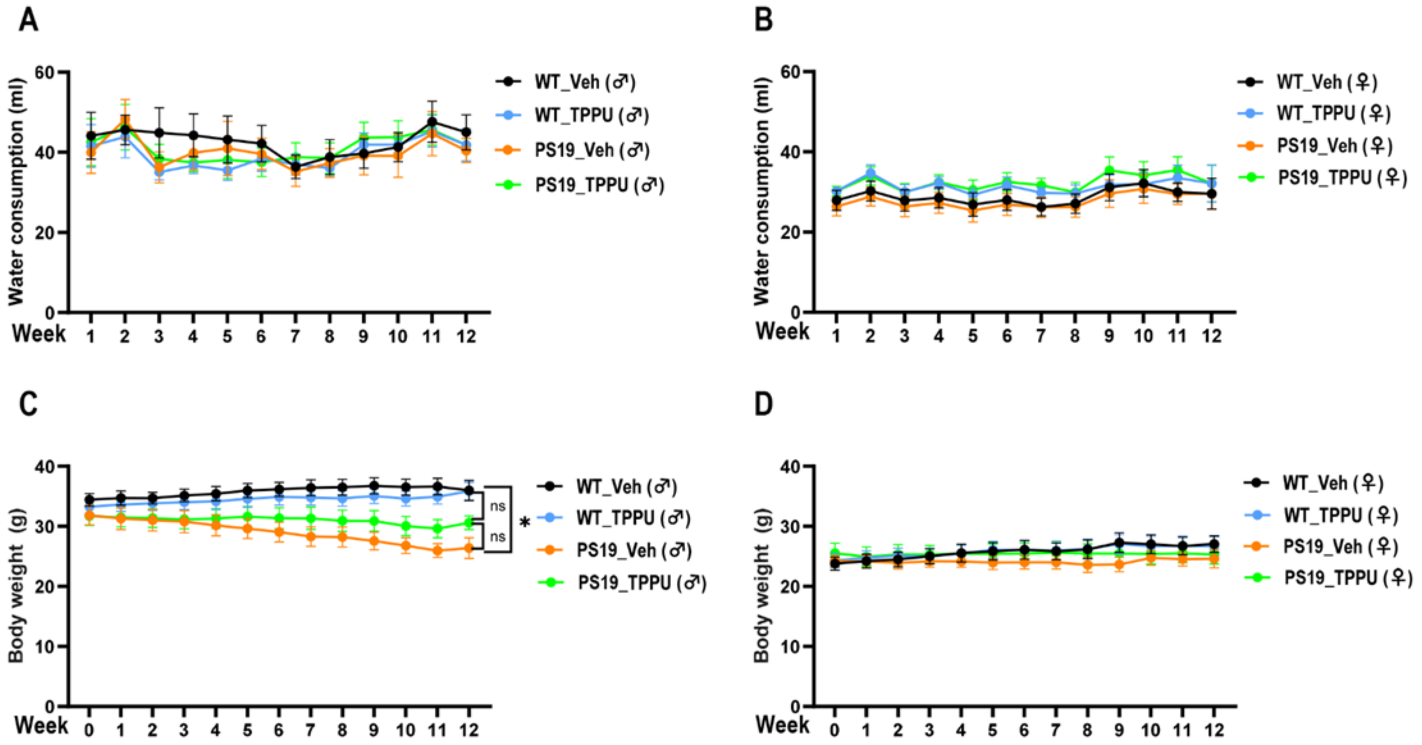

Figure S2. Longitudinal measurement of water consumption (A and B) and body weight (C and D) of WT and PS19 male (A and C) and female (B and D) mice treated with vehicle or TPPU. A: WT_Veh: n=7 cages; WT_TPPU: n=9 cages; PS19_Veh: n=8 cages; PS19_TPPU: n=9 cages. **B:** WT_Veh: n=8 cages; WT_TPPU: n=9 cages; PS19_Veh: n=8 cages; PS19_TPPU: n=9 cages. **C:** WT_Veh: n=10; WT_TPPU: n=12; PS19_Veh: n=9; PS19_TPPU: n=10. **D:** WT_Veh: n=9; WT_TPPU: n=10; PS19_Veh: n=10; PS19_TPPU: n=11. Data are presented as mean ± SEM. Two-way ANOVA with mixed-effects analysis. *$p < 0.05$. All other comparisons non-significant.

Related to Figure 1.

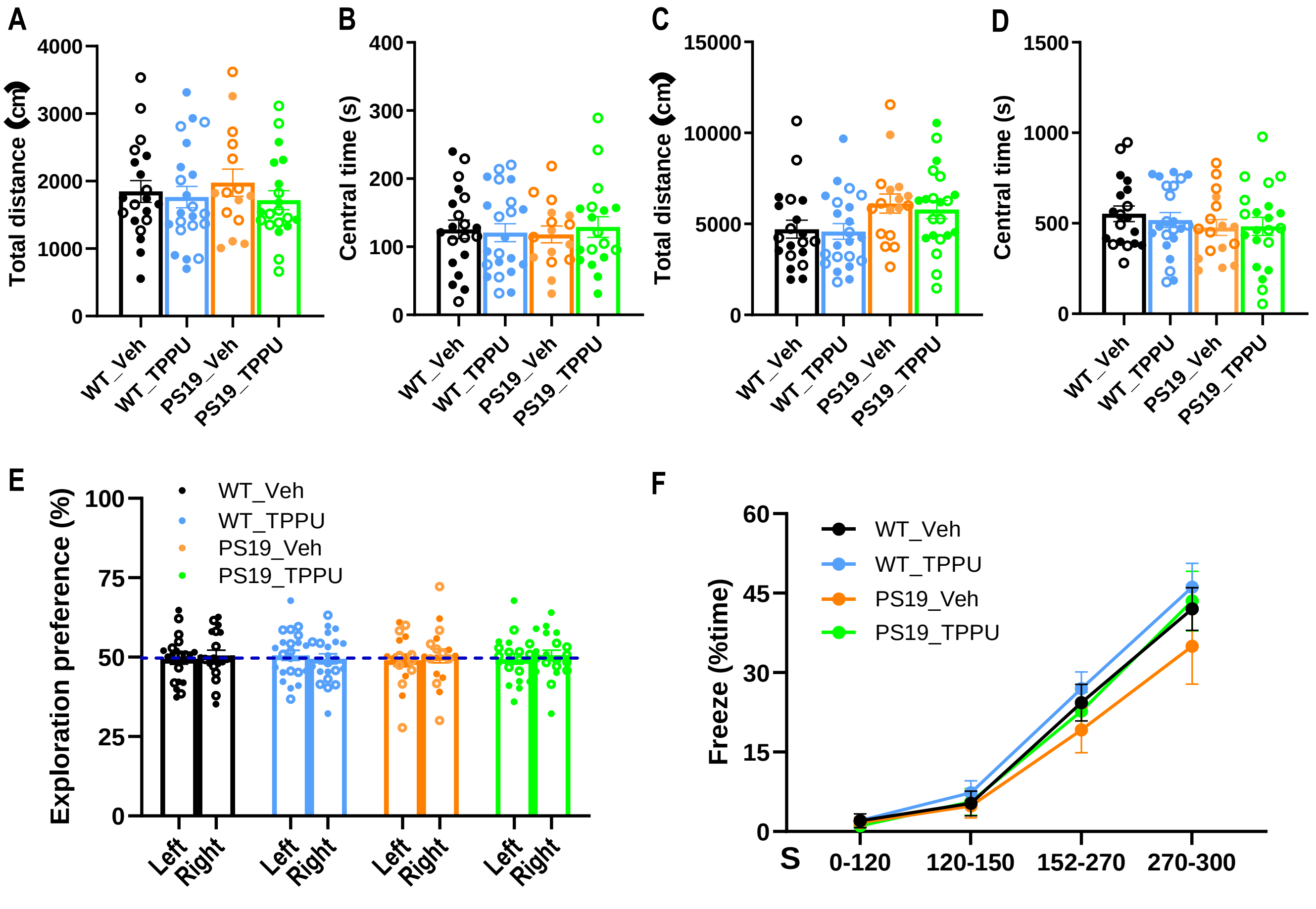

Figure S3. Behavioral assessment of WT and PS19 mice with TPPU treatment.

A & B. Total distance travelled (A) and total time the mice spent in the central area(B) during the first 10 minutes measured in an open field test in WT and PS19 mice treated with vehicle or TPPU. **C & D.** Total distance travelled (C) and total time the mice spent in the central area (D) during the 30 minutes measurement in an open field test in WT and PS19 treated with vehicle or TPPU. WT_Veh: n=11♂ and 9♀; WT_TPPU: n=12♂ and 10♀; PS19_Veh: n=8♂ and 8♀; PS19_TPPU: n=9♂ and 11♀. **E.** Percentage of time exploring the identical object in the left verse right arms during the training phase of NOR for each group. WT_Veh: n=10♂ and 9♀; WT_TPPU: n=12♂ and 9♀; PS19_Veh: n=8♂ and 9♀; PS19_TPPU: n=10♂and 11♀. A-E: Filled circle: male; open circle: female. **F.** Percentage of freezing in different time points during the training phase of fear conditioning test (S: second). WT_Veh: n=11♂ and 9♀; WT_TPPU: n=12♂ and 10♀; PS19_Veh: n=8♂ and 8♀; PS19_TPPU: n=9♂ and 11♀. Data are presented as mean ± SEM. One-way ANOVA with Tukey's multiple comparison test. Non-significant across all comparisons.

Related to Figure 1.

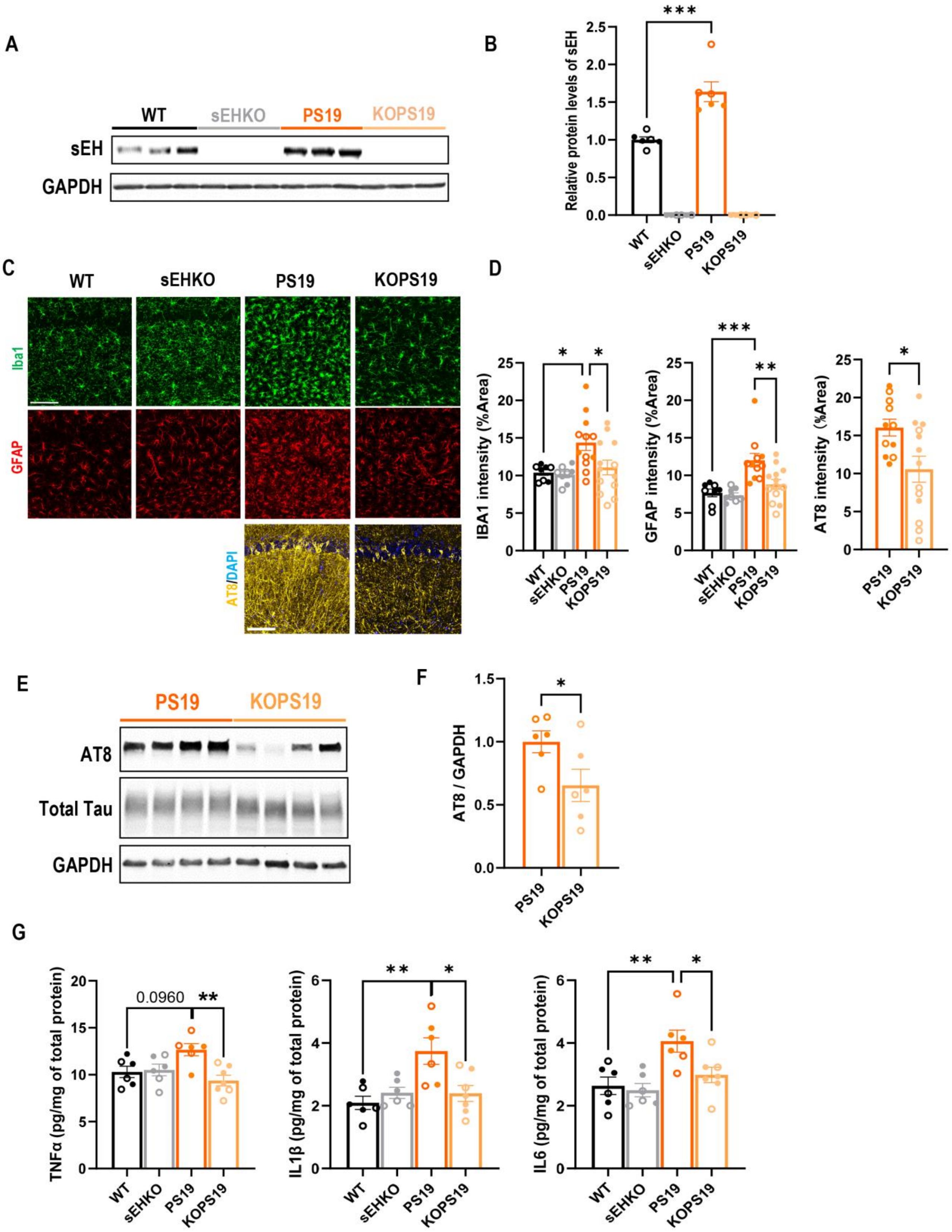

A
WT
sEHKO
PS19
KOPS19
sEH
GAPDH
B
***
Relative protein levels of sEH
WT
sEHKO
PS19
KOPS19
C
WT
sEHKO
PS19
KOPS19
Iba1
GFAP
AT8/DAPI
D
IBA1 intensity (%Area)
GFAP intensity (%Area)
AT8 intensity (%Area)
E
PS19
KOPS19
AT8
Total Tau
GAPDH
F
AT8 / GAPDH
G
TNFα (pg/mg of total protein)
0.0960
IL1β (pg/mg of total protein)
IL6 (pg/mg of total protein)

Figure S4. Reduced Tau pathology and neuroinflammation in PS19 mice by genetic ablation of *Ephx2*.

A & B. Representative Western blot (A) and quantification(B) of WT, *Ephx2*$^{-/-}$ (sEHKO), PS19 and PS19, *Ephx2*$^{-/-}$ (KOPS19) mice using sEH antibody. GAPDH was used as a loading control. n=3♂and 3♀ per group; One-way ANOVA with Tukey's multiple comparison test**. C & D.** Immunofluorescence staining (C) and quantification (D) of PS19 and KOPS19 mice using the AT8, Iba1 and GFAP antibodies. Scale bar: 100µM. Iba1/GFAP staining: WT: n=4♂ and 4♀; sEHKO: n=4♂ and 4♀; PS19: n=5♂ and 7♀; KOPS19: n=7♂ and 6♀. AT8 staining: PS19: n=5♂ and 6♀; KOPS19: n=7♂ and 6♀. One-way ANOVA with Tukey's multiple comparison test. **E & F.** Representative Western blot (E) and quantification(F) of PS19 and KOPS19 mice using AT8 and total Tau antibodies. GAPDH was used as a loading control. n=3♂ and 3♀ per group; Student's *t*-test. **G.** ELISA measurement of proinflammatory cytokines IL1β, IL6 and TNFα in brain lysate of WT, sEHKO, PS19 and KOPS19 mice. n=3♂ and 3♀ for WT, sEHKO, PS19 and n=4♂ and 3♀ for KOPS19. One-way ANOVA with Tukey's multiple comparison test. Filled circle: male; open circle: female. Data is presented as mean ± SEM. *$p < 0.05$; **$p < 0.01$; ***$p < 0.001$.

Related to Figure 2.

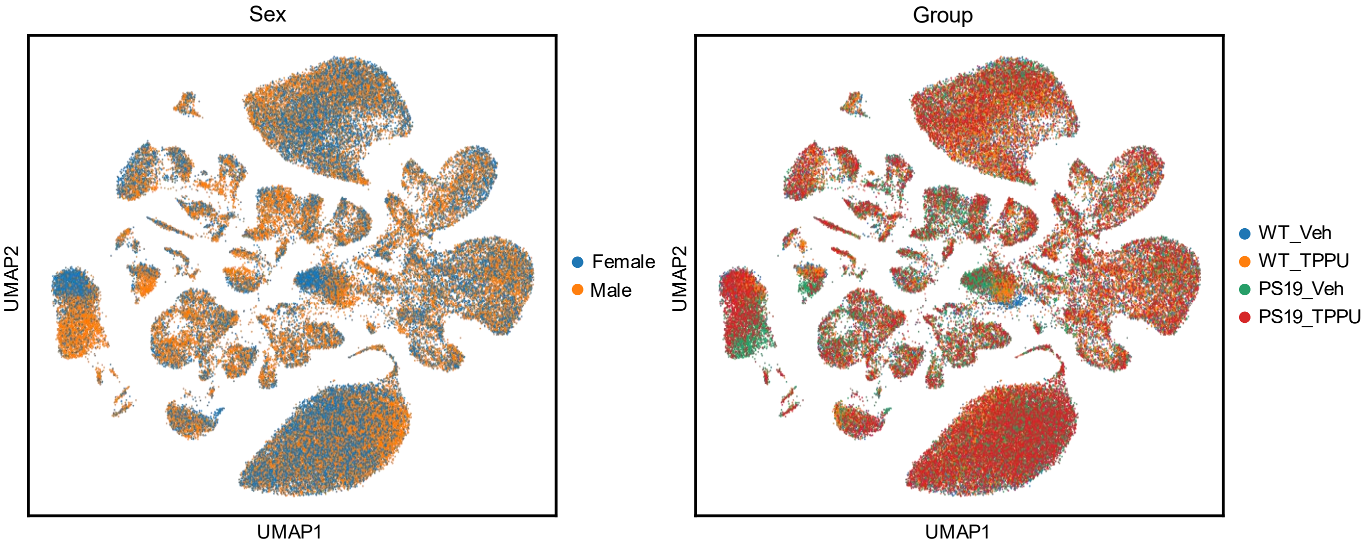

Figure S5. snRNA-seq characterization.

UMAP embedding of snRNA-seq data of 84,226 cells collected from WT and PS19 mice with vehicle or TPPU treatment (2/group) separated by sex (left) and group (right) after batch correction.

Related to Figure 3.

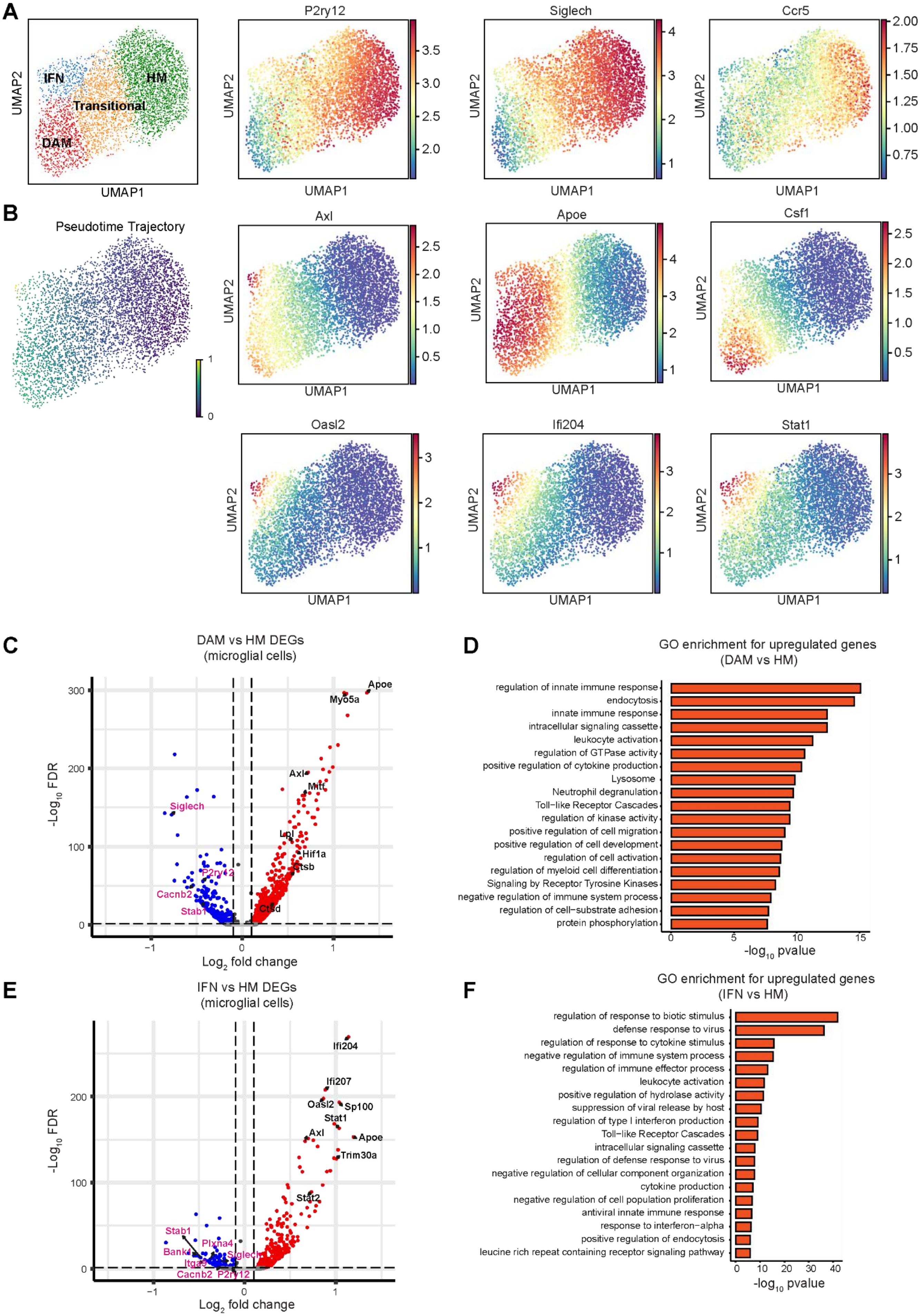
A
UMAP1
UMAP2
IFN
HM
Transitional
DAM
P2ry12
Siglech
Ccr5
B
Pseudotime Trajectory
Axl
Apoe
Csf1
Oasl2
Ifi204
Stat1
C
DAM vs HM DEGs
(microglial cells)
-Log10 FDR
Log2 fold change
Apoe
Myo5a
Axl
Mitf
Lpl
Hif1a
Ctsb
Ctsd
Siglech
P2ry12
Cacnb2
Stab1
D
GO enrichment for upregulated genes
(DAM vs HM)
regulation of innate immune response
endocytosis
innate immune response
intracellular signaling cassette
leukocyte activation
regulation of GTPase activity
positive regulation of cytokine production
Lysosome
Neutrophil degranulation
Toll−like Receptor Cascades
regulation of kinase activity
positive regulation of cell migration
positive regulation of cell development
regulation of cell activation
regulation of myeloid cell differentiation
Signaling by Receptor Tyrosine Kinases
negative regulation of immune system process
regulation of cell−substrate adhesion
protein phosphorylation
-log10 pvalue
E
IFN vs HM DEGs
(microglial cells)
Ifi204
Ifi207
Oasl2
Sp100
Stat1
Axl
Apoe
Trim30a
Stat2
Stab1
Plxna4
Bank1
Itga9
Siglech
Cacnb2
P2ry12
F
GO enrichment for upregulated genes
(IFN vs HM)
regulation of response to biotic stimulus
defense response to virus
regulation of response to cytokine stimulus
negative regulation of immune system process
regulation of immune effector process
leukocyte activation
positive regulation of hydrolase activity
suppression of viral release by host
regulation of type I interferon production
Toll−like Receptor Cascades
intracellular signaling cassette
regulation of defense response to virus
negative regulation of cellular component organization
cytokine production
negative regulation of cell population proliferation
antiviral innate immune response
response to interferon−alpha
positive regulation of endocytosis
leucine rich repeat containing receptor signaling pathway

Figure S6. snRNA-seq analysis of microglial cells.

A. UMAP embedding of snRNA-seq data of microglial subtypes and expression of the marker genes of different microglial subtypes. HM: homeostatic microglia; IFN: interferon response microglia; DAM: Disease-associated microglia. P2ry12, Siglech and Ccr5 for HM; Axl, Apoe and Csf1 for DAM; Oasl2, Ifi204 and Ifi207 for IFN. **B.** UMAP embedding of snRNA-seq data for the pseudo-time trajectory of microglial subclusters. **C.** Volcano plot showing the differentially expressed genes for DAM relative to HM. Upregulated genes are highlighted in red color. Downregulated genes are highlighted in blue color. **D.** GO term pathway enrichment for the upregulated genes in DAM relative to HM. **E.** Volcano plot showing the differentially expressed genes for IFN relative to HM. Upregulated genes are highlighted in red color. Downregulated genes are highlighted in blue color. **F.** GO term pathway enrichment for the upregulated genes in IFN relative to HM.

Related to Figures 4.

Supplemental Table 1: Human demographics (Related to Fig. S1)

Diagnosis	Sex	Age
NCI	F	68
NCI	F	65
NCI	F	67
NCI	F	59
NCI	F	59
NCI	F	65
NCI	M	59
NCI	M	70
NCI	M	70
NCI	M	61
CBD	F	66
CBD	M	66
CBD	M	52
CBD	M	61
CBD	M	44
Picks	M	57
Picks	M	71
Picks	M	72
Picks	M	59
Picks	M	62
PSP	M	70
PSP	M	84
PSP	F	71
PSP	M	71
PSP	F	77

Supplemental Table 2. Summary of snRNA-seq data
(Related to Figs. 3, 4, 5 and Figs. S5 and S6)

Sample	Sex	Cell No (before filtering)	Cell No (after filtering)	Mean UMI	Mean Gene No
WT_Veh_1,2	F	13,348	12,898	7,445	2,623
WT_Veh_3,4	M	14,174	13,579	7,174	2,554
WT_TPPU_1,2	F	11,159	10,740	7,179	2,600
WT_TPPU_3,4	M	11,854	11,405	6,094	2,342
PS19_Veh_1,2	F	2,780	2,747	17,105	3,315
PS19_Veh_3,4	M	13,291	12,947	6,095	2,248
PS19_TPPU_1,2	F	8,935	8,651	8,812	2,832
PS19_TPPU_3,4	M	11,734	11,259	7,899	2,743

Supplementary Table 3. List of Antibodies used in this study

Name	Source	Identifier
Rabbit polyclonal anti-Iba1	FUJIFILM Wako	Cat# 019-19741
Goat polyclonal anti-GFAP	Sigma	Cat# SAB2500462
Chicken polyclonal anti-NeuN	Millipore	Cat# ABN91
Chicken polyclonal anti-Bassoon	Synaptic Systems	Cat# 141 003
Rabbit polyclonal anti-Homer1	Synaptic Systems	Cat# 160 006
Mouse monoclonal AT8	ThermoFisher	Cat# MN1020
Mouse monoclonal MC1	Gifts from Peter Davies	
Mouse monoclonal PHF1		
Rabbit polyclonal anti-Tau	Dako	Cat# A0024
Mouse monoclonal anti-COX2	ThermoFisher	Cat# 35-8200
Rabbit monoclonal anti-synapsin1	Cell Signaling	Cat# 5297
Rabbit polyclonal anti-PSD95	Abcam	Cat# AB18258
Mouse monoclonal anti-PSD95	Synaptic Systems	Cat# 124 011
Chicken polyclonal anti-MAP2	Invitrogen	Cat# PA1-10005
Rabbit monoclonal anti-Synaptophysin	Synaptic Systems	Cat# 101 008
Mouse monoclonal anti-Pan-shank	Millipore	Cat# MABN24
Rabbit polyclonal anti-Grik4	Alomone labs	Cat# AGC-041
Mouse Monoclonal anti-GAPDH	Invitrogen	Cat # AM4300
Mouse Monoclonal anti-β-actin	Proteintech	Cat # 66009
Rabbit anti-human sEH	Gifts from Bruce D. Hammock	
Rabbit anti-mouse sEH		
